# Effects of Synbiotic Supplementation on Bone and Metabolic Health in Caucasian Postmenopausal Women: Rationale and Design of the OsteoPreP Trial

**DOI:** 10.3390/nu16234219

**Published:** 2024-12-06

**Authors:** Alisa Turbić, Liesbeth Vandenput, Anoohya Gandham, Mattias Lorentzon

**Affiliations:** 1 Mary MacKillop Institute for Health Research, Australian Catholic University, Melbourne, VIC 3000, Australia; alisa.turbic@acu.edu.au (A.T.); anoohya.gandham@acu.edu.au (A.G.); 2 Sahlgrenska Osteoporosis Centre, Department of Internal Medicine and Clinical Nutrition, Institute of Medicine, University of Gothenburg, 41345 Gothenburg, Sweden; liesbeth.vandenput@medic.gu.se; 3 Department of Medicine, School of Clinical Sciences at Monash Health, Monash University, Clayton, VIC 3168, Australia; 4 Region Västra Götaland, Department of Geriatric Medicine, Sahlgrenska University Hospital, 43153 Mölndal, Sweden

**Keywords:** osteoporosis, synbiotic, gut microbiota, bone health, metabolic health, postmenopause

## Abstract

Background/Objectives: Correction of decreased diversity of the gut microbiome, which is characteristic of menopause, by supplementation with a synbiotic may attenuate or prevent dysbiosis processes and preserve bone mass. We describe the rationale and design of the OsteoPreP trial aimed at evaluating the effects of 12 months of supplementation with a synbiotic on bone and metabolic health in postmenopausal Caucasian women. Methods: This is a randomized, double-blinded, placebo-controlled trial among 160 Caucasian, postmenopausal women with no current diagnosis of osteoporosis or supplementation with pro- or prebiotics, and no medical treatment affecting bone turnover. Dual-energy X-ray absorptiometry scans will be conducted at screening to confirm absence of osteoporosis. The primary outcome is the relative change (%) in total bone mineral density of the distal tibia at 12 months post-treatment between the active and placebo groups, as determined via high-resolution peripheral quantitative computed tomography. Secondary outcomes are the effects on immune system modulation and cognition, gut microbiota composition, and musculoskeletal and metabolic functions, with particular emphasis on blood glucose regulation. Conclusions: The trial will inform on the efficacy and safety of a synbiotic containing both aerobic and anerobic bacterial strains and a prebiotic fiber on reduction in bone loss and on indices of blood glucose regulation. This trial may pave the way for an exciting field of translational research and be the underpinnings of the prevention strategy of osteoporosis and the management of metabolic dysfunction in postmenopausal women. The trial is registered with clinicaltrials.gov (NCT05348694).

## 1. Introduction

Osteoporosis, a silent, chronic, and multifactorial disorder, is the most common bone disease in humans and is characterized by loss of bone mineral density (BMD) and bone microarchitecture, resulting in decreased bone strength and greater risk of fracture [1]. Despite effective medications that substantially increase BMD and decrease fracture risk, only 30% of those at highest risk are receiving specific osteoporosis treatments in Australia [2]. Fractures of the distal forearm, vertebrae, and hip are the most serious consequences of osteoporosis [3,4]. Hip fractures are linked to an increased risk of mortality and loss of independence, whereas vertebral fractures are linked to significant long-term disability related to pain and kyphosis, leading to increased morbidity and mortality [4,5,6].

The prevalence of osteoporosis and osteoporotic fractures is influenced by various factors, including age, sex, menopausal status, race, and ethnicity. Compared with men, women over 50 years old have a fourfold greater rate of osteoporosis and a twofold greater rate of osteopenia [7]. Osteoporosis affects 200 million women worldwide, with Caucasian and Asian women being more susceptible to osteoporosis than black women [8,9]. This is due to variations in BMD across different racial and ethnic groups [10,11,12]. Osteoporotic fractures affect approximately 1 in 2 women and 1 in 5 men over the age of 50 years [13]. The highest fracture rates are reported among white women, whereas the lowest rates are reported in black women and are approximately 50% lower than those stated in white women [14].

Pharmacological treatments for osteoporosis have been indicated to reduce the risk of fractures by 20–70%, depending on fracture type, in controlled settings but have also proven costly and have been associated with adverse side effects, impacting treatment rates and medication adherence [15,16,17,18,19]. Previous studies in postmenopausal women reported that probiotics increase calcium absorption and 25-hydroxyvitamin D levels and decrease bone loss [20]. Additionally, beneficial effects on glucose regulation in trials investigating the effects of a probiotic or synbiotic (a mixture of probiotics and a prebiotic) intervention in postmenopausal women ranging from 6–12 weeks, including adults diagnosed with type 2 diabetes mellitus (T2DM), have been reported [21,22,23,24]. However, the magnitude of effect reported by these studies was small, perhaps owing to the short duration of these interventions, making it difficult to interpret how clinically meaningful these findings are.

**Figure 1:**
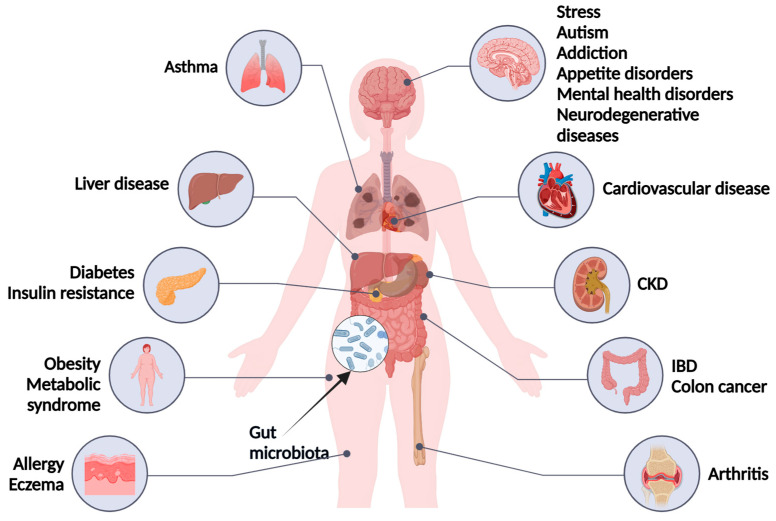
Complications of gut microbiota dysbiosis. CKD = chronic kidney disease; IBD = inflammatory bowel disease.

The risk of osteoporosis in women increases in menopause due to a decrease in estrogen levels, and estrogen-independent mechanisms, including secondary hyperparathyroidism, chronic inflammation, and senility, can add to the development of osteoporosis [25,26,27,28,29]. Moreover, decreased diversity of the gut microbiome in menopause, known as dysbiosis, has been linked to metabolic syndrome, as well as behavioral and physiological deficits (Figure 1) [30,31,32,33]. Metabolic syndrome is a multiplex modifiable risk factor involving several health outcomes, including T2DM, which is normally associated with high body mass index, that share common biological and societal pathways [34,35,36,37,38,39]. T2DM is a worldwide disease affecting 462 million people, and with over 1 million deaths per year attributed to diabetes alone, it is the ninth leading cause of mortality [40]. T2DM cases are equally distributed amongst the genders, with the rate of the disease peaking at about 55 years of age.

The gut microbiota (GM) plays an important role in the maturation of the immune system, and both the GM and the immune system can regulate bone health [21,27,31,32,41,42], and varies with race, sex and ethnicity [43,44,45,46]. Postmenopausal women with a diagnosis of osteoporosis have also been reported to have a significantly different GM composition and diversity than those with normal bone mass with the genus *Prevotella histicola* being more prevalent in those with normal bone mass, suggesting its protective effects on bone health [47]. Probiotics, more specifically the regulatory role of short chain fatty acids (SCFAs) has been linked to maintaining an equilibrium in the GM by ensuring bone health is controlled by the immune system, and the potential of probiotic and prebiotic supplementation to stimulate SCFA production has been reported [48,49]. The SCFA butyrate (C4) has been identified as a major regulator of osteoclast metabolism and bone homeostasis by promoting the expansion of T regulatory (Treg) cells [50,51]. GM, through the production of SCFAs, has also been demonstrated to play important roles in regulating physiological functions, including blood glucose regulation, inflammation, brain function and behavior [32,52,53,54]. For example, it has been suggested that SCFAs generated from the gut microbiota regulate the number and function of regulatory T cells in the colon, thereby influencing the development of inflammation and associated diseases such as hypertension [55].

There is increasing evidence for an association of dysbiosis, or an imbalance in the gut microbiota, with psychiatric and cognitive dysfunction, suggesting the gut-brain axis is critical for the management of these conditions [56,57,58,59]. Gut microbiota, through the production of important neurotransmitters, has been demonstrated to play important roles in modulating the activity of the brain through immune pathways, which directly or indirectly guides social behavior [54,60,61]. As neuroinflammation is critically linked to indices of cognitive decline, investigating the potential benefits of increasing SCFAs could be particularly beneficial for the aging population.

Given the critical role of probiotics in several processes central to osteoporosis prevention, a unique opportunity exists to examine the safety and efficacy of this relatively inexpensive, readily available, oral dietary supplement in a clinical trial focused on preventing osteoporosis. The trial intervention is a formulation of five probiotic strains important for the production of SCFA butyrate, which have previously been found to be beneficial to bone and metabolic health and neurodevelopmental disorders [62,63,64,65,66,67]. Additionally, the intervention contains a prebiotic, inulin, known to promote the growth of beneficial microbes and/or promote beneficial changes in the activity of the microbiome [68]. To our knowledge, this is the first randomized controlled trial to administer a mix of all five probiotic strains and a prebiotic in a supplement formulation (Pendulum WBF-038) to human subjects with a risk of developing osteoporosis, over a 12-month treatment period. If supplementation with the trial synbiotic is beneficial for bone health, cardiometabolic health, muscle health, and cognition, the results of this trial could translate into significant improvements in the prevention of osteoporosis and related musculoskeletal and metabolic conditions, such as T2DM [69].

## 2. Materials and Methods

### 2.1. Trail Design

**Figure 2:**
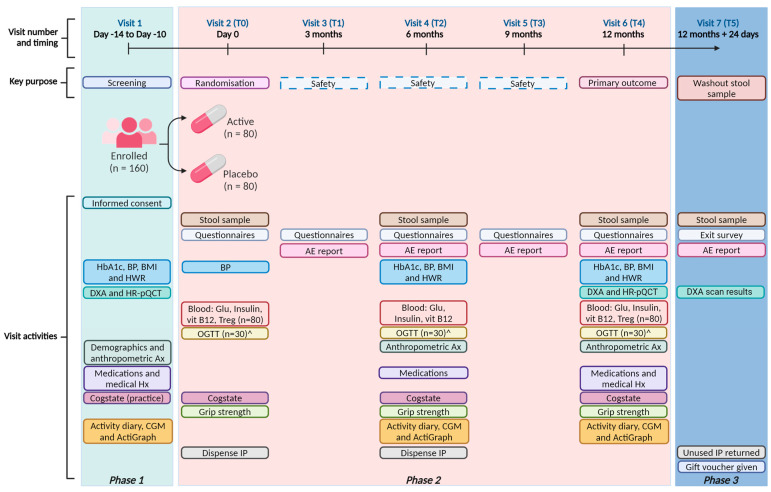
Summary of OsteoPreP trial visits and activities. AE = adverse event; Ax = assessment; Hx = history; IP = investigational product; HbA1c = glycated hemoglobin; BP = blood pressure; BMI = body mass index; HWR = height-to-weight ratio; DXA = dual-energy X-ray absorptiometry; HR-pQCT = high-resolution peripheral quantitative computed tomography; CGM = continuous glucose monitor; Glu = glucose; vit B12 = vitamin B12; Treg = T regulatory cell; OGTT = oral glucose tolerance test; ^ = musculoskeletal subgroup.

The trial is a randomized, double-blinded, placebo-controlled trial. Participants will be assessed at baseline (T0) upon recruitment and subsequently at five follow-up visits. The trial will be conducted in three, phases as summarized in Figure 2 and described in detail in Supplementary File S1.

**Figure 3:**
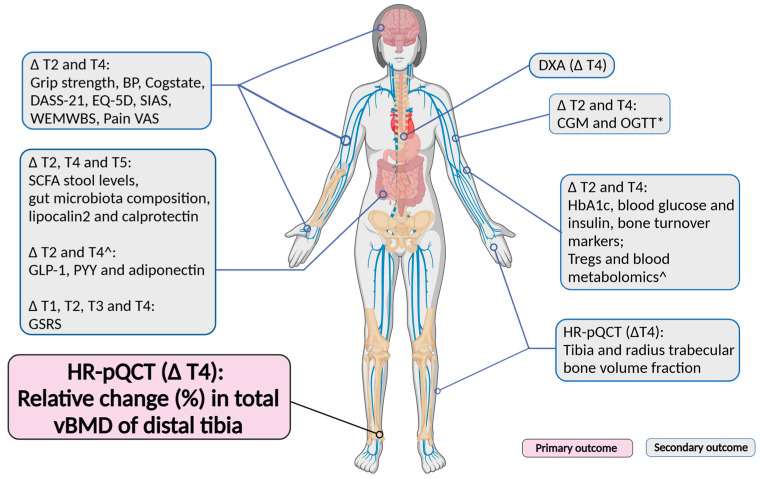
Summary of OsteoPreP trial primary and secondary outcomes. BP = Blood pressure; DASS-21 = Depression Anxiety Stress Scale-21; EQ-5D = EuroQOL Five Dimensions quality of life scale; SIAS = Social Interaction Anxiety Scale; WEMWBS = Warwick-Edinburgh Mental Wellbeing Scale; Pain VAS = Visual Analogue Scale for pain; BMD = Bone Mineral Density; DXA = Dual-energy X-ray Absorptiometry; HR-pQCT = High-Resolution Peripheral Quantitative Computed Tomography; CGM = Continuous Glucose Monitor; OGTT = Oral Glucose Tolerance Test; HbA1c = Hemoglobin A1C blood sugar (glucose) level test; ^ = musculoskeletal subgroup at twelve months only; SCFA = Short Chain Fatty Acids; GLP-1 = Glucagon-Like Peptide-1 receptor agonist; GSRS = Gastrointestinal Symptom Rating Scale; * = musculoskeletal subgroup will undergo OGTT.

Dual-energy X-ray absorptiometry (DXA) scans will be conducted on participants at screening. Following this, participants will be assigned randomly to either a synbiotic supplement or a matching oral placebo arm. The efficacy of the supplement will be established by comparing data collected after 12 months of treatment with those collected at T0. The study outcomes are illustrated in Figure 3. Only Caucasian women will be recruited in this trial owing to significant differences in BMD and bone remodeling between ethnicities and races.

The trial will be advertised on social media platforms, relevant public locations, and newspapers, and through assisted mail out of trial information conducted by a government agency to community-dwelling women aged 40–65 years residing in the inner suburbs of Melbourne, Victoria, Australia.

### 2.2. Participants and Recruitment Process

**Figure 4:**
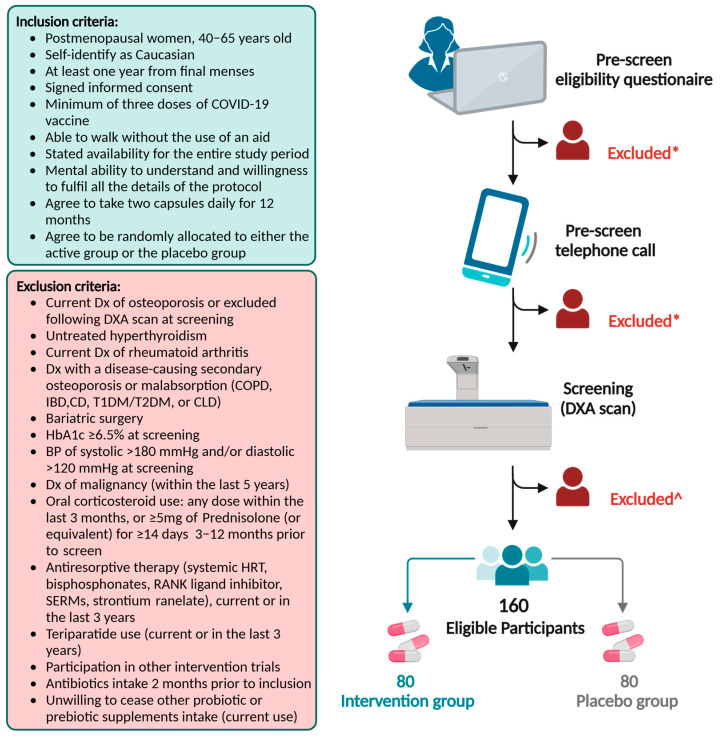
OsteoPreP trial inclusion and exclusion criteria and screening process. * Do not meet the inclusion criteria; ^ = DXA, BP, or HbA1c ineligible participant; Dx = diagnosis; DXA = dual-energy X-ray absorptiometry; COPD = chronic obstructive pulmonary disease; IBD = inflammatory bowel disease; CD = celiac disease; T1DM = type 1 diabetes mellitus; T2DM = type 2 diabetes mellitus; CLD = chronic liver disease; HbA1c = hemoglobin A1C blood sugar (glucose) level test; BP = blood pressure; HRT = hormone replacement therapy; RANK = receptor activator of NF-κB ligand; SERMs = selective estrogen receptor modulators.

One hundred and sixty 40–65-year-old postmenopausal women will be recruited. Women interested in the trial will complete an online prescreening questionnaire, as advertised in the trial brochure. Further screening for eligibility will be conducted by trained research staff during telephone interviews. During this call, trial procedures and consent forms, including trial aims, requirements, participants’ right to withdraw, and any potential risks involved, will also be discussed. Eligible participants will be enrolled in the trial provided that no exclusion criteria are met at the time of screening, including the presence of osteoporosis, high blood pressure (BP), and diabetes. Women with identified osteoporosis following DXA scans will be informed of their exclusion from the trial as well as the rationale for exclusion and referred to their primary care physician. The trial inclusion and exclusion criteria are illustrated in Figure 4.

### 2.3. Randomization Process

Participants will be randomized at the T0 visit and within 14 days of enrollment. A block randomization (80 blocks; 2 participants per block) will be used to allocate participants to one of the two study groups. Additionally, randomization will involve alternating blocks, with 50% of participants (40 blocks) allocated to have their regulatory T lymphocyte counts measured. The musculoskeletal subgroup data analysis comprises the first 30 participants (15 in each arm) who consent to the additional optional assessment of an oral glucose tolerance test. The regulatory T cells of participants in the musculoskeletal subgroup will be counted.

A central randomization schedule will be generated by the company (Pendulum Therapeutics Inc., San Francisco, CA, USA) providing the investigational product (IP) Pendulum WBF-038. The sponsor, as well as an independent external statistician, will hold the key to unblind the trial at its conclusion. The study IP will be provided to the researchers pre-coded and blinded to the trial group, where each bottle is uniquely coded according to the randomization list. The randomization schedule will be programmed into an electronic case report form system, which is housed on a Research Electronic Data Capture (REDCap) platform [70]. The participants will be randomized at a 1:1 ratio to either the intervention or placebo arm.

Unless there is an emergency, trial IP randomization codes will be accessible only after all data for each participant have been entered into the trial database and the database has been finalized. Should an emergency case arise where the investigator decides that a participant cannot be adequately treated without knowing the identity of their treatment allocation, the coding will be disclosed. To break a study code, the investigator will ask the company or the external statistician to provide the supplement group allocation. If any unblinding occurs prior to the trial conclusion, the time, date, study code, and justification for unblinding will be documented.

### 2.4. Interventions

All bacteria contained in the IP are commensal organisms that are known to inhabit the human gastrointestinal tract under normal conditions. Pendulum WBF-038 is a proprietary synbiotic formulation of five human-derived bacterial strains: *Akkermansia muciniphila* WB-STR-0001, *Anaerobutyricum* (formerly *Eubacterium*) *hallii* WB-STR-0008, *Clostridium butyricum* WB-STR-0006, *Clostridium beijerinckii* WB-STR-0005, and *Bifidobacterium infantis* Bi26TM, plus chicory inulin and magnesium stearate. The bacterial strains were grown in an FDA-registered food facility adhering to current good manufacturing practice conditions for food manufacturing facilities. Pendulum WBF-038 contains no genetically modified organisms, and its bacterial strains are generally recognized as safe.

The IP will be provided to the research team as acid-resistant capsules in identical bottles, which have been pre-coded by the supplier according to the randomization list and stored refrigerated at 4 °C. The participants received either Pendulum WBF-038 or matched placebo capsules containing only magnesium stearate. The daily dose in two capsules of WBF-038 is ≥2 × 10^9^ active fluorescent units. The dosage was determined in reference to a previous trial [71]. At T0, the participants will be dispensed IP along with calendars to record their adherence as well as a tip sheet on how to take the IP (Figures S1 and S2). The participants will be directed to take the assigned IP twice daily within 30 min of the morning and evening meals during phase 2 (treatment) of the trial. At six (T2), twelve (T4), and final (T5) visits, participants will be instructed to return the used IP containers (and any unconsumed IP), and compliance will be recorded. Compliance will be calculated based on returned capsules and the information recorded by participants on their IP calendars, with >80% compliance required for continuation in the study. The IP intake will be discussed at each visit to ensure compliance is tracking as per protocol. If a participant is unable to take the allocated IP because of an adverse event (AE), the researchers will alter the principal investigator according to internal guidelines.

### 2.5. Restrictions During Participation in the Trial

Participants will be instructed not to take other products containing probiotic bacteria, to fast overnight (water is allowed and encouraged) before visits, which require the collection of blood samples, and to refrain from hormone replacement therapy for the duration of the trial. Should a participant withdraw early from the trial, they will not be unblinded before the data analysis is complete. The participants will be requested to continue their normal physical activity and dietary patterns while in the trial.

### 2.6. Visit Windows

The T0 visit date will set the follow-up visit dates, which will be booked one week on either side of the designated visit date unless there are extenuating circumstances (for example, unavailable due to illness). Any visit/contact that occurs outside one week on either side of the visit windows will be recorded in REDCap as a protocol deviation for that visit. Visits requiring blood sampling and stool collections will be conducted six months apart at T0, T2, and T4, with an additional stool collection at the final visit. Additionally, participants will be given a 72-h window in which to collect the stool samples and all materials required for the collection. Visits requiring fasted blood collection will be scheduled early in the morning and at a time convenient for study participants. The screen and T4 visits, which include DXA and HR-pQCT scans, will take no longer than 3 h to complete. Participants will be provided with breaks, food, and drinks at all in-person visits, as needed. To ensure adherence, participants will be sent email reminders of what a visit entails a week prior to each visit and contacted the day before the visit via telephone, where requirements for the visit will be discussed again.

### 2.7. Outcomes

#### 2.7.1. Primary Outcome

High-resolution peripheral quantitative computed tomography (HR-pQCT) scans will be performed to assess total BMD (BMD) and bone microstructure at the nondominant distal radius and distal tibia. Scans will be obtained at the standard ultradistal site and 30% of the bone length of the tibia and radius sites (XtremeCT II, Scanco Medical AG, Brüttisellen, Switzerland) with a protocol described earlier [72]. Once images are processed, the following parameters are obtained: total BMD (mg/cm^3^), cortical cross-sectional area (mm^2^), cortical BMD (mg/cm^3^), and the trabecular bone volume fraction (%). The muscle area, density, intramuscular fat and subcutaneous fat, at the distal tibia will also be measured via HR-pQCT. The CVs for the HR-pQCT bone measurements in our facility range from 0.29% to 3.61% for the tibia bone measurements.

#### 2.7.2. Secondary Outcomes

##### Dual-Energy X-Ray Absorptiometry (DXA)

Areal bone mineral density (aBMD, g/cm^2^) will be measured at the lumbar spine (L1–L4) and proximal femur (total hip and femoral neck) as well as the full body composition (fat and lean mass) via Lunar iDXA, a GE HealthCare machine with Lunar iDXA enCORE Bone & Metabolic Health software version 18 (GE HealthCare, Buckinghamshire, UK). Standardized procedures for participant positioning and scan analysis will be used [73]. Follow-up measurements will be evaluated via the ‘comparison’ feature of the Lunar iDXA enCORE Bone & Metabolic Health software Version 18. DXA is widely recognized as the gold standard for evaluating BMD and is routinely used for diagnosing both primary and secondary osteoporosis [73]. DXA derived BMD data will be used as part of both the exclusion criteria and outcome measures (secondary) at screening and T4 visits, respectively. The percentage coefficient of variation (CV) for scans repeated on 30 participants in our facility is 0.63% for the lumbar spine, 1.18% for the femoral neck aBMD, and 0.69% for the whole-body BMD.

##### Continuous Glucose Monitoring (CGM)

The participants will be fitted with the minimally invasive Freestyle Libre Pro IQ CGM at the screen, T2 and T4 visits per the manufacturer’s instructions [74], and they will wear the device for a duration of 10 days. This system, a commercially available factory-calibrated sensor, is waterproof and is inserted rather painlessly into the subcutaneous tissue over the triceps. This device measures interstitial glucose continuously in the range of ≥40 mg/dL to 500 mg/dL [74]. By measuring interstitial glucose every 5–15 min, the device provides a comprehensive 24-h glycemic profile, with enhanced assessment of nocturnal and/or asymptomatic hypoglycemia and pattern recognition during the treatment period.

##### Physical Activity Monitoring

To assess habitual physical activity and sedentary behavior, participants will be fitted with a triaxial ActiGraph GT3x accelerometer (ActiGraph, Pensacola, FL, USA) worn at the hip (Figure S3). Before the visit, the accelerometer will be initialized by research staff to record data at a sampling frequency of 30 Hz in three axes: vertical, mediolateral, and anteroposterior, using ActiLife software (V6.13.4 Lite Edition, ActiGraph, FL, USA). The device will be fitted at screen, T2, and T4, and worn for a 10-day period. The data will be downloaded and aggregated into 1-min epochs via ActiLife software (V6.13.4 Lite Edition, ActiGraph, FL, USA). At these visits, participants will also receive a diary to record the times they wear an accelerometer, their sleep and wake times and their physical activity levels (Figure S4). The participants will be encouraged to maintain their normal daily routines during this period.

##### Trial Questionnaires

The questionnaires, either self-reported or obtained in a semi-interview with the trial participants, will be administered via and recorded in REDCap. Table 1 shows an overview of the trial questionnaires and the associated timepoints for data collection. Two in-house self-report questionnaires will be administered. Specifically, dietary patterns of fermented food intake will be measured via an exploratory self-reported fermented food frequency questionnaire and an exit survey, both of which are administered via REDCap (Supplementary File S4).

##### Blood Biomarker Measurements

There will be three venous fasting blood collection timepoints (T0, T2, and T4) and three finger prick blood draws (screening, T2, and T4). Blood samples will be collected from the cubital vein by a qualified venipuncturist at a hospital pathology contracted by the researchers.

At T0, T2, and T4, blood samples will be tested for fasting blood glucose and insulin by the hospital’s pathology laboratory staff via a commercial enzymatic kit (Beckman Coulter Inc., Brea, CA, USA) with a CV of 1.6–2.6% and a chemiluminescent microparticle immunoassay Architect Insulin 8K41 kit (Abbott GmbH & Co., Wiesbaden, Germany) with a precision of ≤7% total CV. The remaining serum and plasma will be aliquoted and stored in a −80 °C freezer for further analysis. At the completion of the trial, these samples will be analyzed via commercially available immunosorbent assays for the following bone turnover markers: C-terminal cross-linking telopeptide of type I collagen, procollagen type 1 N-terminal propeptide, high-sensitivity C-reactive protein and osteocalcin at T0, T2, and T4. A flowchart of the blood collection and processing procedure is illustrated in Figure S5.

Furthermore, the first 80 randomized participants will undergo an additional blood analysis of the number of circulating regulatory T cells (Tregs) (n = 40 per arm). Blood for this analysis will be collected at T0 and T4, and this collection will not require an additional blood draw. Tubes will be spun by pathology staff before being sent by a courier to a collaborator for peripheral blood mononuclear cell isolation and storage in liquid nitrogen until completion of the trial [91]. Treg analysis will be subcontracted to an external facility at the completion of the trial.

##### CogState Brief Battery

Cognitive performance will be measured via the CogState brief battery for outcome purposes at screen (practice), T0, T2, and T4 [92]. Practice will assist in controlling the learning effect associated with the assessment. CogState has been specifically designed to detect changes in cognitive performance from repeated measures in a clinical trial setting. This tool demonstrates good acceptability, efficiency, and stability and minimizes practice effects. The test battery will include the Groton maze learning test (executive function test), the continuous paired associate learning test (spatial paired associative learning test), the one back test (working memory test), and the social-emotional cognition test (emotional recognition) [93,94,95]. The total administration time will be approximately 24 min.

##### Hand Grip Strength (HGS)

At T0, T2, and T4, participants will complete an assessment of their HGS as measured by the amount of static force individuals can generate with one hand via a Jamar digital dynamometer (Patterson Medical, Kenora, ON, Canada) with a standardized protocol [96]. HGS measurements are reliable when standardized methodology and calibrated equipment are utilized. The participants will be instructed to sit in a chair holding the dynamometer with a 90-degree angle at the elbow. The dynamometer’s position two (counted from the display) of five possible settings will be used. Three measurements will be collected for each hand, resulting in a total of six measurements alternating between the right and left hands. A trained researcher will conduct the assessment.

##### Stool Sample Collection, Storage and Analysis

Stool sample kits, a Bristol Stool Form Scale (BSFS), and instructions will be provided to the participants at the screen visit. BSFS, with its accompanying illustrations, was developed in 1997 and has become a widely used practical guide to classify stools into seven groups on the basis of the evidence that the appearance of stools is dependent on the length of time that it spends in the colon, as reported previously [97]. The participants will collect the samples at home within the preceding 24–72 h of their T0, T2, T4, and T5 visits and indicate their stool type on collection containers as per BSFS, the date, and the time of stool passage. The samples will be stored at −20 °C until they are ready to be brought back. Stool samples will be shipped to an external laboratory for processing and analyses of SCFAs, probiotic strain deoxyribonucleic acid (DNA), and the gut microbiome using 16s and metagenome sequencing to verify intake of the IP by the intervention group. The samples will be run in triplicate.

##### Oral Glucose Tolerance Test (OGTT)

A further subset of participants (15 per arm, 30 in total) will be invited to participate in a musculoskeletal subgroup, involving an additional assessment of a 2-h OGTT at T0, T2, and T4 [98,99]. Blood samples will be collected into tubes containing ethylenediaminetetraacetic acid for the analysis of glucose, glucagon-like peptide-1 receptor agonist, peptide YY, and adiponectin (Figure S6) at the completion of the trial. The samples are shipped to an external facility for analysis.

### 2.8. Sample Size

The sample size calculation was based on data from a previous probiotic intervention study in older women, which demonstrated a significant effect on bone health outcomes. For this study, additional metabolic outcomes are included, and effect sizes for these outcomes were estimated based on prior research using similar synbiotic interventions in comparable populations.

Permitting for possible participant attrition, the total sample size for the trial will be 160 (80 per arm), taking a 20–30% drop-out rate into account. Statistical power analysis will be performed in relation to the primary outcome of the relative twelve-month change (%) in total BMD using HR-pQCT at the distal tibia. On the basis of a similar probiotic intervention in older women [100], the expected decrease in tibia BMD is 1.85% in the placebo arm and 0.9% decline at most in the treatment arm, with a standard deviation (SD) of 1.6% and an alpha of 0.05; 46 participants are needed in each arm to achieve >80% power. To be able to detect smaller groups to group differences and to account for an attrition rate of 20–30% and due to many secondary outcomes, we will recruit a minimum of 80 participants into each arm.

### 2.9. Statistical Analysis

All outcomes and analyses will be prospectively categorized as primary, secondary, or exploratory. Categorical variables will be described by numbers and percentages and 95% confidence intervals (CIs) for percentages based on a binomial distribution. Continuous variables will be described by means, SDs, medians, minima, and maximums, and 95% CIs for the means. The main comparisons will be of the intervention vs. placebo. With respect to dichotomous variables, Fisher’s exact test will be used to compare between the two groups. For continuous variables, Fisher’s nonparametric permutation test will be used.

To address the concern regarding multiple comparisons, the statistical analysis plan will include methods to control for Type I error across the primary and secondary outcomes. The primary variables will be investigated as the percentage difference from T0 to T4 visit between the two study groups. The trial will be deemed positive if the intervention vs. placebo is significant at the 0.05 significance level. The primary variable is anticipated to be normally distributed and analyzed by applying analysis of covariance (ANCOVA) with the relative change from T0 to T4 as the dependent variable, the treatment group as the fixed effect, and the T0 value as the covariate. The least square means with 95% CIs for the active treatment group vs. the placebo group will be presented together with the associated *p*-value.

The secondary variables are continuous and possibly will not be normally distributed. For secondary outcomes, adjustments will be made using appropriate correction methods, such as the Bonferroni adjustment for family-wise error control or the Benjamini–Hochberg procedure for controlling the false discovery rate, depending on the degree of correlation between outcomes. This approach ensures the integrity of the analyses while accounting for the diversity of outcomes assessed in the study.

Primary and secondary analyses will be conducted for the intention-to-treat as well as the per-protocol population. The difference in parameter estimates between the intervention and the placebo will be assessed for its clinical relevance. The secondary analyses will be considered exploratory, and an alpha of 0.05 will be applied with no further adjustments.

Participants who supply both T0 and end-of-treatment samples will be included in a secondary dose–response analysis to account for early trial withdrawals or inadequate compliance.

## 3. Results

The trial is currently underway, and as such, results are not yet available.

## 4. Discussion

The development of evidence-based, safe, and acceptable prevention strategies at the population level to target multiple risk factors for osteoporosis, T2DM, and metabolic syndrome in women and the population in general, and to reduce the health and economic burden of the condition, is warranted. Osteoporosis and associated fractures are an increasing global public health concern, with the current practice being predominantly aimed at secondary prevention following fractures, rather than primary prevention at the community level [101]. Similarly, the burden of metabolic diseases such as T2DM is increasing globally, and a rate higher in developed regions, including Western Europe, warranting urgent public health and clinical preventive measures [40].

Recent trials focusing on supplementation of postmenopausal women with probiotics have reported that it may increase BMD, with the evidence for it being stronger in women with osteopenia than osteoporosis. A randomized trial of three months of supplementation with *Bifidobacterium animalis* subsp. *lactis* amongst 40 postmenopausal women reported no significant change in BMD, but co-administering the probiotic with conventional drugs was found to be beneficial in managing postmenopausal osteoporosis, suggesting a beneficial mechanism of probiotic adjunctive treatment [102]. Moreover, a significant decrease in bone turnover markers following daily consumption of *Lactobacillus acidophilus* for three months in a randomized trial of 55 postmenopausal women has been reported [103]. Similarly, Vanitchanont et al. examined a three-month supplementation of 40 osteopenic postmenopausal women with a multispecies probiotic [104]. reported a significant decrease in the serum bone resorption marker (C-terminal telopeptide of type I collagen) compared to baseline and suggested that supplementation with multispecies probiotics may have a preventive effect on bone through their antiresorptive effects. Though these studies are limited by a small number of participants and short intervention periods, we note a study conducted by Glogowska-Szelag et al. amongst 172 postmenopausal women [105]. They described a decrease in T-score of the lumbar spine measured by DXA following 12 months in the placebo but not the probiotic group, suggesting that the oral administration of a mix of two species of *Lactiplantibacillus* may be a viable strategy for prevention of BMD loss.

This trial is the first RCT to investigate the effects of Pendulum WBF-038 for the prevention of osteoporosis and improvement of metabolic health following a 12-month treatment period. This trial is designed to: (i) provide greater insight of the link between the GM and bone health; (ii) evaluate the effectiveness of the effects of a dietary supplement in altering the gut microbiome to improve bone and metabolic health in postmenopausal women and consequently reduce or eliminate the risk factors for metabolic syndrome, including high blood pressure, high blood glucose, and a large waistline; (iii) investigate the potential effect of the trial intervention on immune system modulation as a potential mediator; and (iv) increase the capability for community-based health care professionals to implement evidence-based and probiotic-based therapies for the prevention and management of osteoporosis and T2DM management. It is expected that the findings of this trial will provide scientific evidence for the use of synbiotic supplements in the primary care of postmenopausal women at risk of developing osteoporosis as well as in the management of T2DM patients.

## Figures and Tables

**Table 1:** Summary of the OsteoPreP trial questionnaires.

Questionnaire	Collection Method	Data Collection Timepoint						
		Screen	Baseline T0	3 Months T1	6 Months T2	9 Months T3	12 Months T4	Final * T5
Demographic data	SSI	X						
Medical History	SSI	X					X	
Current Medications	SR and SSI	X	X	X	X	X	X	X
Risk of future fracture	In person via FRAX^®^	X					X	
IPAQ-SF	SR		X		X		X	
Dietary surveys (DQES v3.2, FFFQ, GSRS)	SR		X	^a^ X	X	^a^ X	X	
Mental health and wellbeing surveys (SIAS, WEMWS, EQ-5D-5L, Pain VAS, DASS-21)	SR		X		X		X	

FRAX^®^ = The Fracture Risk Assessment Tool adopted for the Australian population [75]. IPAQ-SF = International Physical Activity Questionnaire-Short Form [76]. DQES v3.2 = Dietary Questionnaire for Epidemiological Studies [77,78,79]. FFFQ = Fermented Food Frequency Questionnaire. GSRS = Gastrointestinal Symptom Rating Scale [80,81]. DASS-21 = Depression, Anxiety, and Stress Scale-21 [82,83,84]. SIAS = Social Interaction Anxiety Scale [85,86]. WEMWS = Warwick-Edinburgh Mental Wellbeing Scale [87,88]. EQ-5D-5L = EuroQol Five Dimensions quality of life scale [89,90]. Pain VAS = Pain Visual Analogue Scale [90]. X = Signifies a timepoint at which questionnaire is administered. * T5 visits occur 24 days post 12 months (T4) visit. ^a^ Only GSRS surveys will be administered at T1 and T3. SSI = Semi-structured Interview. SR = Self-reported.

## Data Availability

The protocol is available from the corresponding author upon request.

## Supplementary Materials

The following supporting information can be downloaded at: https://www.mdpi.com/article/10.3390/nu16234219/s1, File S1: The OsteoPreP trial phases; File S2: OsteoPreP trial Investigational Product (IP) instructions, Figures S1 and S2; File S3: ActiGraph GT3x accelerometer management and physical activity diary, Figures S3 and S4; File S4: OsteoPreP trial in-house surveys; File S5: OsteoPreP trial pathology flowchart, Figure S5; File S6: OGTT test blood collection flowchart, Figure S6. File S7: OsteoPrep trial—Participant Information Letter; File S8: OsteoPreP mechanistic sub-study—Participant Information Letter. Reference [71] are cited in the Supplementary Materials.
