# Supplementary material for: Effects of Synbiotic Supplementation on Bone and Metabolic Health in Caucasian Postmenopausal Women: Rationale and Design of the OsteoPreP Trial
Source: Nutrients. 2024 Dec 6;16(23):4219. doi: 10.3390/nu16234219 (PMC11644401; doi:10.3390/nu16234219)
Supplement: Supplementary file 1 [file nutrients-16-04219-s001.zip › nutrients-3338017-supplementary/Supplementary files/Supplementary file S1.pdf]

Supplementary File S1. The OsteoPreP trial phases

Phase 1. Screen visit

Evidence for criteria fulfilment will be based on participants' reports obtained prior to the visit. Enrolment informed consent (see Additional file 7) will be obtained from the participants, and eligibility will be further assessed. Women who agree to participate in the additional musculoskeletal subgroup analysis will be asked to sign an additional, separate consent form (see Additional file 8). A finger prick blood sample will be taken to measure glycated haemoglobin A1c (HbA1c) using the Cobas b 101 (Roche Diagnostic, USA), and resting BP will be measured using an Omron Digital Blood pressure monitor HEM-907 (Omron Healthcare Co., Japan) from the nondominant arm via an automated oscillatory device. Women who have an HbA1c ≥ 6.5% (48 mmol/mol) and systolic BP >180 mmHg and/or diastolic BP >120 mmHg will be excluded from the trial and provided a letter of their results and relevant fact sheets and are recommended to contact their general practitioner (GP). Women will be asked to take off their jacket and shoes for height (cm) and weight (kg) assessments, using a stadiometer (Livingstone International, Australia; precision 0.1 cm; range 0–200 cm) and an electronic scale (Seca 877, Seca Ltd, Birmingham, UK; precision 100 g; range 2–200 kg), respectively after which their body mass index will be will be calculated as body mass (kg) divided by the height (m) squared. Waist circumference (cm) will be measured between the lowest rib and the top of the hip bone directly over the skin, yet not by compressing the skin, after breathing out normally. Hip circumference will be measured around the widest portion of the buttocks. Waiste and hip measurements will be undertaken by a trained researcher using a tape measure (Livingstone International, Australia; precision 0.1 cm; range 0–150 cm).

Participants' areal bone mineral density (aBMD, g/cm2) will be measured at the lumbar spine (L1-L4) and proximal femur (total hip and femoral neck) using Hologic Lunar Dual-energy X-ray absorptiometry (iDXA) GE HealthCare machine with Lunar iDXA enCORE Bone & Metabolic Health

software version 18 (GE HealthCare, United Kingdom). DXA derived BMD data will be used as part of both the exclusion criteria and outcome measures (secondary) at screening and T4 visits, respectively. If the participant's scan results indicate no sign of osteoporosis at the screen visit, their scan will also be utilised for the outcome measures, including the BMD of the lumbar spine and hip as well as the full body composition (fat and lean mass).

Women with identified osteoporosis, as defined by a T score of -2.5 or less at the femoral neck or lumbar spine (L1-L4), will be excluded at the screening visit and informed of the findings. They will be provided a letter from the research team indicating the results of the scan and an osteoporosis fact sheet, and they will be encouraged to see a GP for further investigations. At this point they will also be informed that they are not eligible to continue in the trial owing to the scan results. Those women who have a clear DXA scan (no osteoporosis) at the screen visit will undergo a high-resolution peripheral quantitative computed tomography (HR-pQCT) scan where the volumetric BMD (vBMD) and bone microstructure at the nondominant distal radius and the distal tibia at the nondominant limbs will be investigated.

Following the completion of the bone scans, the researcher will conduct a semistructured interview with the participant to obtain information regarding their medical history and demographics and ask them to complete a familiarisation CogState testing session. The participants will then be fitted with a minimally invasive Freestyle Libre Pro IQ Continuous Glucose Monitor (CGM) (Abbott Diabetics Care, UK) and asked to wear it for 10 days in addition to wearing an ActiGraph GT3x accelerometer (ActiGraph, FL, USA) with a belt at the right hip. During this time, participants will also be asked to complete a physical activity diary and record their sleep schedules. Finally, they will be asked to collect a stool sample (no more than 72 hour-old) prior to their T0 visit, for which all required materials will be provided. After the visit, the participants will be emailed a calendar invitation with instructions for how to prepare for the baseline (T0) visit and links to relevant online questionnaires to complete within

one week prior to the visit. Participants who meet the inclusion criteria will be randomised within two weeks after the screen visit.

Phase 2. Treatment

At the T0 visit, participants will be randomly assigned to one of the two groups. They will arrive in an overnight fasted state and bring their T0 stool sample, accelerometer, activity diary and CGM. They will undergo a series of assessments, including BP assessment, a fasting blood sample, a CogState assessment, and a hand grip strength (HGS) assessment. Those women (n=30) further randomised to the mechanistic subgroup will complete a two-hour OGTT. At the end of this visit, participants will be provided with their six-month supply of the investigational product (IP).

One week prior to the three (T1) and nine (T3) month visits, a trained researcher will activate the Gastrointestinal Symptom Rating Scale questionnaire in the trial database for participants to complete before the visit and message them to organise a time suitable for the telephone visit. At these visits, participants will be assessed for any AEs (which may be related or unrelated to trial participation) and compliance.

Prior to the six month (T2) visit, participants will be asked to prepare in much the same way as for the T0 visit. Assessments at this visit will be as at screen and baseline visits, but no bone scans will be performed, and an assessment of IP compliance and AEs will be conducted. At the end of the visit, participants will be given a reply paid envelope to return CGM, an accelerometer and a trial activity diary after 10 days. At this timepoint, participants will also be provided with their next six months supply of IP.

Twelve month (T4) visit will involve conducting the same protocol as at the T2 visit with the addition of DXA and HR-pQCT scans, and a medical history check. Following this visit participants will

continue to take the IP for an additional 10 days (while they wear a CGM device and an accelerometer). They will also be instructed to collect the final stool sample at two weeks from when they take the last dose of the trial IP, and a date for the final (T5) visit will be determined.

Phase 3. Washout period (minimum two weeks) and final visit

At the T5 participants will drop off the stool samples they collected at least two weeks after the last dose of the trial IP along with the CGM device, accelerometer, activity diary, IP compliance calendars, and IP containers. This timepoint was established to investigate whether the IP was present in the stool (washed out). Upon completion of the final visit, all participants will be invited to complete an online exit survey asking tFhem for their feedback about their experiences in taking part in the OsteoPreP trial.
