# Supplementary material for: Effects of Synbiotic Supplementation on Bone and Metabolic Health in Caucasian Postmenopausal Women: Rationale and Design of the OsteoPreP Trial
Source: Nutrients. 2024 Dec 6;16(23):4219. doi: 10.3390/nu16234219 (PMC11644401; doi:10.3390/nu16234219)
Supplement: Supplementary file 1 [file nutrients-16-04219-s001.zip › nutrients-3338017-supplementary/Supplementary files/Supplementary file S2.pdf]

Supplementary File S2: OsteoPreP trial Investigational Product (IP) instructions

IP will be dispensed at baseline (T0) and six months (T2) visits. Participants will receive 7 bottles (60 capsules/bottle) at T0 and an additional 6 bottles at T2 visit. At T0 participants will also be given a Pendulum Therapeutics Inc. FAQs tip sheet (Figure S1 and S2) and follow the instructions therein, and calendars with fridge magnet to track their intake of the IP. Participants will be informed that symptoms such as transient mild nausea, loose stools, and diarrhea during the initial 3–5 days of use are common [1], and to inform the research team should symptoms persist or worsen. Participants will be asked to return the used supplement containers and unconsumed supplements at the T2 and 12-month (T4) visits for compliance check.

FREQUENTLY ASKED QUESTIONS

Pendulum® OsteoPreP Study

How should I take the capsules?

Take 2 capsules daily with food, 1 capsule in the morning and 1 capsule in the evening.

Should I take the capsules with food or on an empty stomach?

Take 1 capsule, twice a day, with food. Taking capsules on an empty stomach may cause gastrointestinal distress.

Can I take the capsules at the same time as other medications and supplements?

Yes.

What should I do if I missed a dose?

If you miss your morning dose, or if you don't eat food in the morning, take 1 capsule with lunch and 1 capsule with dinner. Or take 2 capsules with dinner, as tolerated.

If you miss your evening dose, take it with food, as soon as you remember. For the capsules to work the best, they should be taken with food.

I missed my dinner dose, should I take 3 capsules the next day?

We do not recommend taking 3 capsules at once, or in the same day, as this may cause gastrointestinal distress.

How should I store the bottle?

Store it in a cool and dry place. The refrigerator is recommended.

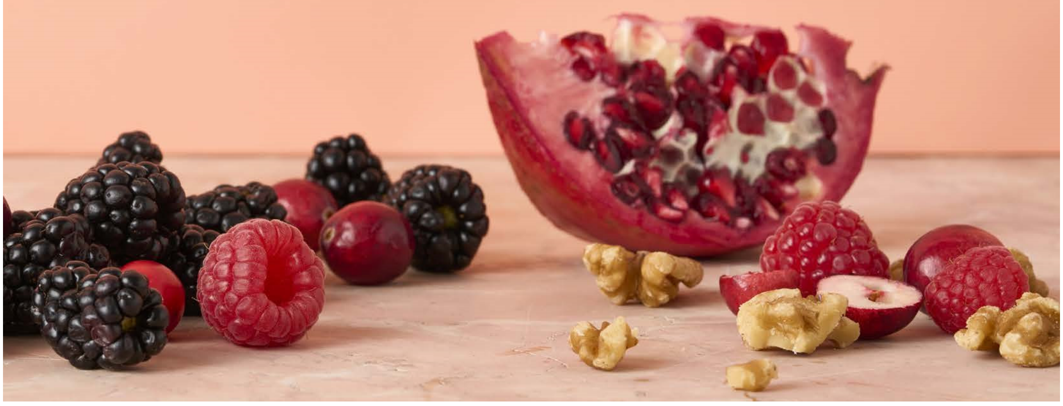

Figure S1. Instructions for IP intake and storage given to participants at baseline visit (page 1).

FREQUENTLY ASKED QUESTIONS

Pendulum® OsteoPreP Study

Can I travel with the capsules?

Yes. Keep the capsules in the bottle or remove the capsules you will need and carry them in a sealed container or plastic bag to minimize heat and moisture exposure. Upon arrival at your destination, store them in a refrigerator.

Storing the capsules in carry-on luggage or a purse is okay as long as the duration is no longer than 7 days and there is no exposure to direct sunlight or high temperatures. If refrigeration is not available for more than 7 days, then store the capsules in an insulated container with ice packs.

Why shouldn't I load the capsules into my pill organizer with my other supplements?

Using a weekly pill organizer is not recommended because moisture entering through gaps and moisture from surrounding pills may compromise the capsules.

I'm going out to eat, can I bring the capsule with me at room temperature?

Yes. Short periods of time at room temperature is okay and should not impact the product. Remember to keep capsules in a sealed container to prevent any moisture exposure and avoid direct sunlight.

My capsules are not all the same color. Is this safe?

Yes. Occasionally, capsules may contain more food coloring than others, causing color variation. There is absolutely no impact regarding food safety. The food coloring we use is made from fruit and vegetable juice.

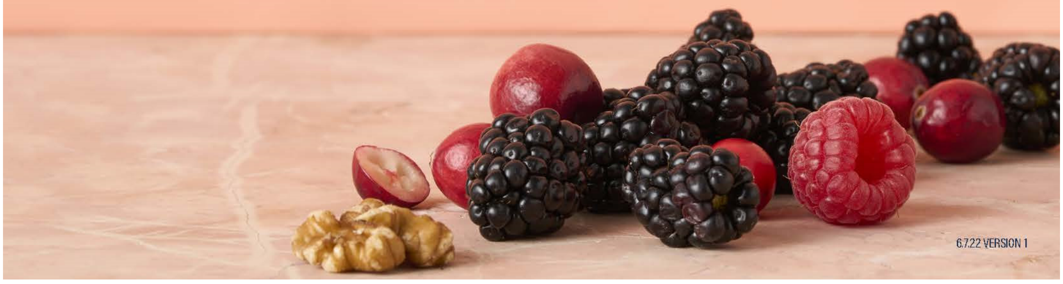

Figure S2. Instructions for IP intake and storage given to participants at baseline visit (page 2).

1. Perraudeau F, McMurdie P, Bullard J, Cheng A, Cutcliffe C, Deo A, et al. Improvements to postprandial glucose control in subjects with type 2 diabetes: a multicenter, double blind, randomized placebo-controlled trial of a novel probiotic formulation. BMJ Open Diabetes Res Care. 2020;8(1). 10.1136/bmjdrc-2020-001319.
