# Supplementary material for: Effects of Synbiotic Supplementation on Bone and Metabolic Health in Caucasian Postmenopausal Women: Rationale and Design of the OsteoPreP Trial
Source: Nutrients. 2024 Dec 6;16(23):4219. doi: 10.3390/nu16234219 (PMC11644401; doi:10.3390/nu16234219)
Supplement: Supplementary file 1 [file nutrients-16-04219-s001.zip › nutrients-3338017-supplementary/Supplementary files/Supplementary file S3.pdf]

Supplementary File S3. ActiGraph GT3x accelerometer management and physical activity diary

The accelerometers will be synchronized to the clock of the computer used in the trial, with the correct participant trial number assigned, and the correct way of wearing the accelerometer and matters needing attention will be explained to the participants. The device will be fitted at screen, six (T2) and twelve-months (T4) visits, and worn for a 10-day period, as illustrated in Figure S3. Participants will be instructed to wear an accelerometer at the right hip, while removing it for showering, water sports and sleeping. Participants will be asked to show research staff how they would fit the device on themselves as confirmation of proper wear. The device will provide no activity feedback to participants.

At these visits, participants will also be provided with a diary to record the time of day the accelerometer is worn and removed, their sleep and wake times, and physical activity and any issues that may have influenced physical activity estimates (e.g. health issues resulting in low activity) and be asked to go about their normal daily routines. The physical activity diary will contain 10 identical pages (one for each day an accelerometer is worn), (Figure S4).

Participants will return the device and diary in person at baseline (T0) and T4 visits, and by post 10 days after T2 visit in a pre-paid envelope provided.

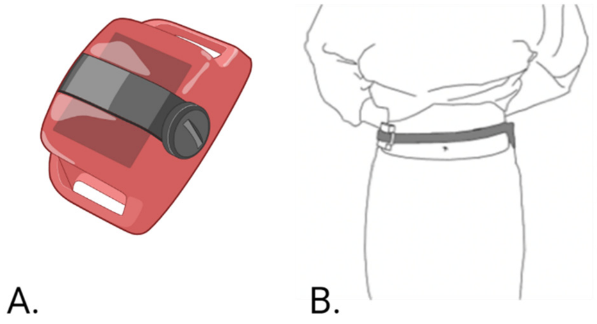

Figure S3. ActiGraph GT3x accelerometer positioning. A) An image of an ActiGraph GT3x accelerometer device; B) An image of where on the body ActiGraph GT3x accelerometer is worn by participants.

Activity logs

For each day that you wear the physical activity monitor, we will ask you to keep a log:

1. Start by writing the **date and day of the week** in the top row.
2. Please record **when** you put your **monitor** on. If you **remove the monitor for more than 10 minutes**, please indicate the time(s) that you took the monitor off and put it back on. Again, this is particularly important as we cannot tell if you are not wearing it, or if you are lying down.
3. Please record the time, duration(s), and intensity of any structured physical activity you completed.

*Structured physical activity includes exercise such as attending the gym, organised sport, etc.

Day of week: **Date:**

Sleep (previous night)

Please try and complete these questions within an hour of getting out of bed in the morning.

1. What time did you try to go to sleep? ___:___ am / pm
2. How long did it take you to fall asleep? _______ min
3. How many times did you wake up, not counting final awakening? _________ time(s)
4. In total, how long did these awakenings last? ______ min
5. What time was your final awakening? ______:___ am / pm
6. What time did you get out of bed for the day? _______:___ am / pm
7. Rate quality of sleep (circle one): Very poor Poor Fair Good Very good

Activity monitor

Did you remove your activity monitor during the day for >15 minutes?

☐ **No** ☐ **Yes** (see below)

Removed at...	***Back on at...***
___:___ am / pm	___:___ am / pm
___:___ am / pm	___:___ am / pm
___:___ am / pm	___:___ am / pm

Activity Log

If you completed any structured physical activity today, please list the details separately below

Time ___:____ AM / PM Duration _____ min
Type ________________________________
Intensity ____________________________

Time ___:____ AM / PM Duration _____ min
Type ________________________________
Intensity ____________________________

Time ___:____ AM / PM Duration _____ min
Type ________________________________
Intensity ____________________________

☐ Please tick if you did not complete any structured physical activity today

Figure S4. An example of OsteoPreP trial physical activity diary given to participants at screen, six and twelve month visits.
