# Supplementary material for: Effects of Synbiotic Supplementation on Bone and Metabolic Health in Caucasian Postmenopausal Women: Rationale and Design of the OsteoPreP Trial
Source: Nutrients. 2024 Dec 6;16(23):4219. doi: 10.3390/nu16234219 (PMC11644401; doi:10.3390/nu16234219)
Supplement: Supplementary file 1 [file nutrients-16-04219-s001.zip › nutrients-3338017-supplementary/Supplementary files/Supplementary file S4. OsteoPreP trial in-house surveys.pdf]

FFFQ

Please complete the survey below.

Thank you!

The following questions are about the amount of fermented food you have eaten IN THE LAST MONTH.

In the last month, how often did you eat a serve of the following foods?

---

1) 1. Vinegars: apple cider vinegar, white/red wine vinegar/ white vinegar, malt vinegar?

(1 tablespoon = 1 serve)

- ◯ Never
- ◯ Less than once a month
- ◯ 1 to 3 times per month
- ◯ 1 time per week
- ◯ 2 times per week
- ◯ 3 to 4 times per week
- ◯ 5 to 6 times per week
- ◯ 1 time per day
- ◯ 2 times per day
- ◯ 3 or more times per day

---

2) 2. Pickled foods: pickled onions, pickles?

(1 whole item = 1 serve)

- ◯ Never
- ◯ Less than once a month
- ◯ 1 to 3 times per month
- ◯ 1 time per week
- ◯ 2 times per week
- ◯ 3 to 4 times per week
- ◯ 5 to 6 times per week
- ◯ 1 time per day
- ◯ 2 times per day
- ◯ 3 or more times per day

---

3) 3. Fermented vegetables: sauerkraut, kimchi?

(1 tablespoon = 1 serve)

- ◯ Never
- ◯ Less than once a month
- ◯ 1 to 3 times per month
- ◯ 1 time per week
- ◯ 2 times per week
- ◯ 3 to 4 times per week
- ◯ 5 to 6 times per week
- ◯ 1 time per day
- ◯ 2 times per day
- ◯ 3 or more times per day

---

4) 4. Potatoes, pasta, rice or oats that have been cooked and cooled before being eaten cold?

(1/2 cup = 1 serve)

- ◯ Never
- ◯ Less than once a month
- ◯ 1 to 3 times per month
- ◯ 1 time per week
- ◯ 2 times per week
- ◯ 3 to 4 times per week
- ◯ 5 to 6 times per week
- ◯ 1 time per day
- ◯ 2 times per day
- ◯ 3 or more times per day

5) 5. Fermented soy products: tempeh, miso?

(Tempeh: 75 g [size of a deck of cards] = 1 serve)
(Miso: 1 tablespoon = 1 serve)

- ◯ Never
- ◯ Less than once a month
- ◯ 1 to 3 times per month
- ◯ 1 time per week
- ◯ 2 times per week
- ◯ 3 to 4 times per week
- ◯ 5 to 6 times per week
- ◯ 1 time per day
- ◯ 2 times per day
- ◯ 3 or more times per day

6) 6. Parmesan cheese or cultured butter?

(40 g Parmesan cheese [matchbox size] or 25 g [1 tablespoon] cultured butter = 1 serve)

- ◯ Never
- ◯ Less than once a month
- ◯ 1 to 3 times per month
- ◯ 1 time per week
- ◯ 2 times per week'
- ◯ 3 to 4 times per week
- ◯ 5 to 6 times per week
- ◯ 1 time per day
- ◯ 2 times per day
- ◯ 3 or more times per day

7) 7. A serve of cooked legumes: lentils, chickpeas, beans, or peas, for example?

(1/2 cup cooked legumes = 1 serve)

- ◯ Never
- ◯ Less than once a month
- ◯ 1 to 3 times per month
- ◯ 1 time per week
- ◯ 2 times per week
- ◯ 3 to 4 times per week
- ◯ 5 to 6 times per week
- ◯ 1 time per day
- ◯ 2 times per day
- ◯ 3 or more times per day

8) 8. Under ripe/green bananas?

(1 banana = 1 serve)

- ◯ Never
- ◯ Less than once a month
- ◯ 1 to 3 times per month
- ◯ 1 time per week
- ◯ 2 times per week
- ◯ 3 to 4 times per week
- ◯ 5 to 6 times per week
- ◯ 1 time per day
- ◯ 2 times per day
- ◯ 3 or more times per day

The following questions are about the amount of fermented drinks that you have drunk

9) 9. In the last month, how many glasses of Kombucha (fermented tea) did you usually drink each day?

- ◯ 250mL = 1 glass
- ◯ 375mL can = 1.5 glasses
- ◯ 750mL bottle = 3 glasses
- ◯ 1.25L bottle = 5 glasses
- ◯ 1.5L bottle = 6 glasses
- ◯ 2L bottle = 8 glasses
- ◯ Do not drink Kombucha

10) 10. In the last month, how many glasses of kefir (a fermented milk drink) did you usually drink each day?

- ◯ 250mL = 1 glass
- ◯ 375mL can = 1.5 glasses
- ◯ 750mL bottle = 3 glasses
- ◯ 1.25L bottle = 5 glasses
- ◯ 1.5L bottle = 6 glasses
- ◯ 2L bottle = 8 glasses
- ◯ Do not drink kefir

Exit survey

Please complete the survey below.

Thank you!

Thank you for participating in the OsteoPreP Study.

We would like your feedback to better understand your participation experience. Please complete this short exit survey.

Thank you!
OsteoPreP Team

What were your treatment expectations of participating in this study?

____

Please list any benefits you experienced by participating in this study

____

Did you experience any unintended effects from participating in this study?

____

How tolerable did you find the supplements to take?

____

How tolerable did you find the dose of supplements you were asked to take?

____

While participating in the study, could you tell you were on a treatment?

◯ Yes
◯ No

If yes, why/how?

____

Do you have any feedback or comments regarding the procedures you where asked to undergo?

◯ Yes
◯ No

If so, what?

____

Do you have any feedback or comments regarding the questionnaires you were asked to complete?

◯ Yes
◯ No

If so, what?

____

Do you have any feedback or comments regarding the study's research team?	◯ Yes<br>◯ No
If so, what?	__________________________
Would you like to receive a notification of the findings of the project?	◯ Yes<br>◯ No
If so, what is your preferred method of notification?	◯ Email<br>◯ Post
