# Supplementary material for: Effects of Synbiotic Supplementation on Bone and Metabolic Health in Caucasian Postmenopausal Women: Rationale and Design of the OsteoPreP Trial
Source: Nutrients. 2024 Dec 6;16(23):4219. doi: 10.3390/nu16234219 (PMC11644401; doi:10.3390/nu16234219)
Supplement: Supplementary file 1 [file nutrients-16-04219-s001.zip › nutrients-3338017-supplementary/Supplementary files/Supplementary file S5.pdf]

Supplementary File S5: OsteoPreP trial pathology flowchart.

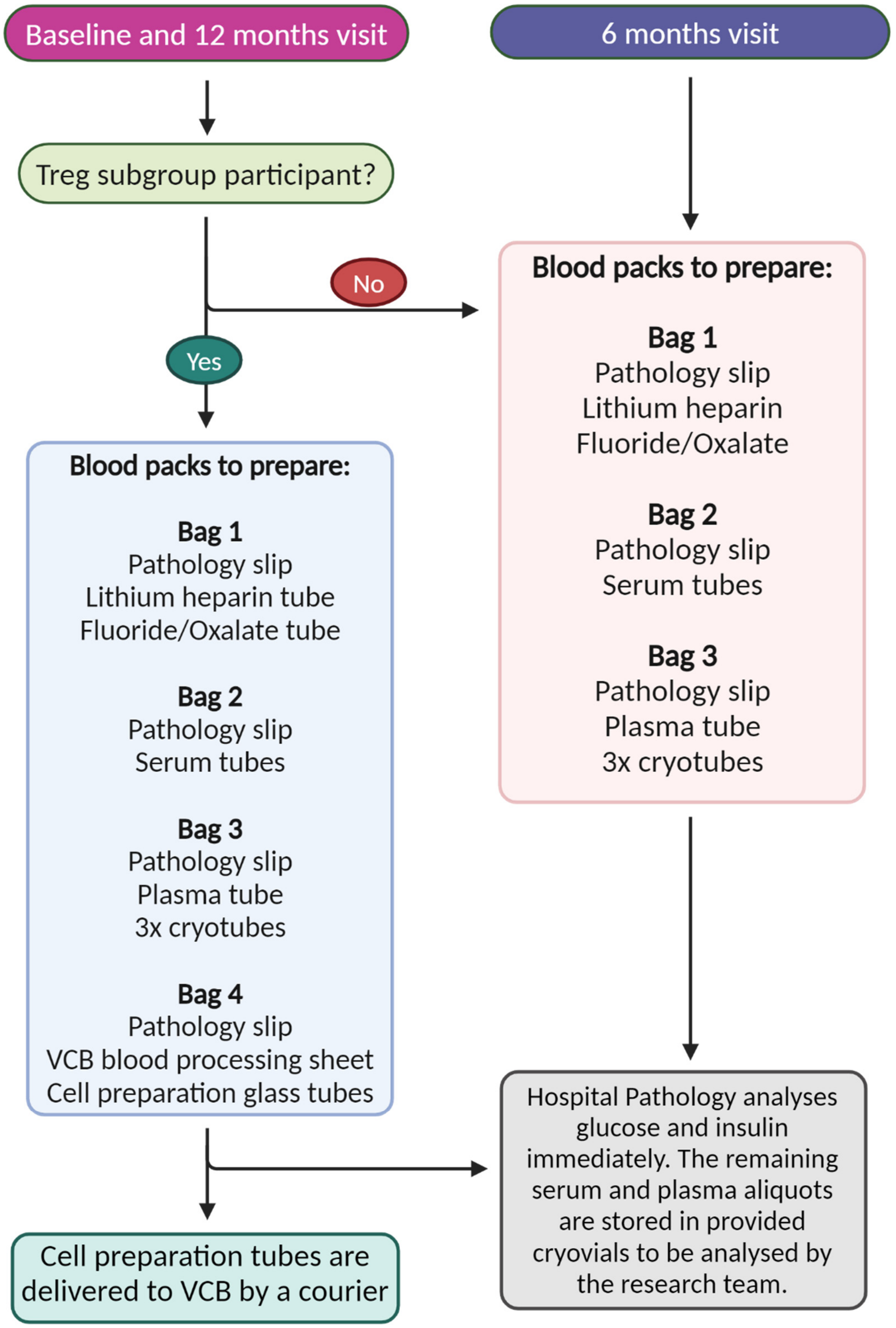

Figure S5. OsteoPreP trial pathology blood collection and processing flowchart.

Tregs = regulatory T cells; PBMCs = peripheral blood mononuclear cells; VCB = Victorian Cancer Biobank
