# Supplementary material for: Effects of Synbiotic Supplementation on Bone and Metabolic Health in Caucasian Postmenopausal Women: Rationale and Design of the OsteoPreP Trial
Source: Nutrients. 2024 Dec 6;16(23):4219. doi: 10.3390/nu16234219 (PMC11644401; doi:10.3390/nu16234219)
Supplement: Supplementary file 1 [file nutrients-16-04219-s001.zip › nutrients-3338017-supplementary/Supplementary files/Supplementary file S6.pdf]

Additional File S6: Blood sample collection for oral glucose tolerance test (OGTT)

Musculoskeletal subgroup participants (n=30) will undergo an OGTT test at baseline, six and twelve months visits. A trained researcher will carry out the test and explain the procedure to the participant. Test will be conducted while the participant is in a fasted state. Figure S6 illustrates blood tubes collected at each test timepoint. Sample will be analysed by a hospital pathologist contracted by the researchers.

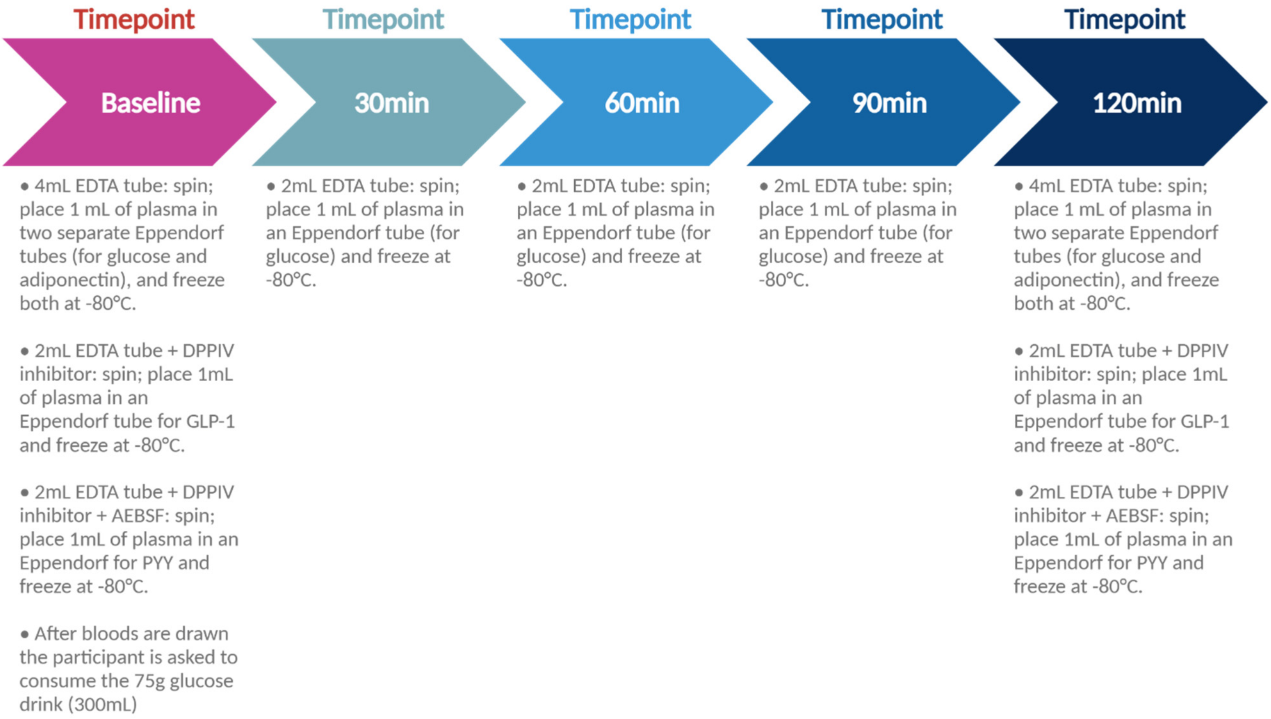

Figure S6. OGTT test blood collections and processing flowchart conducted at baseline, six and tweleve months for musculoskeletal subgroup participants.
