# Supplementary material for: Effects of Synbiotic Supplementation on Bone and Metabolic Health in Caucasian Postmenopausal Women: Rationale and Design of the OsteoPreP Trial
Source: Nutrients. 2024 Dec 6;16(23):4219. doi: 10.3390/nu16234219 (PMC11644401; doi:10.3390/nu16234219)
Supplement: Supplementary file 1 [file nutrients-16-04219-s001.zip › nutrients-3338017-supplementary/Supplementary files/Supplementary file S7.pdf]

AUSTRALIAN CATHOLIC UNIVERSITY

PARTICIPANT INFORMATION LETTER

PROJECT TITLE: OsteoPreP: The effect of probiotic supplementation on bone, muscle, and glucose metabolism in postmenopausal women: A randomized placebo-controlled trial

APPLICATION NUMBER: 2021-122HC

PRINCIPAL INVESTIGATOR: Prof John Hawley

Dear Participant,

You are invited to participate in the research project described below.

What is the project about?

Many health benefits are linked to the good bacteria in our gut. Just as the bacteria help us, we can help them by eating certain types of food supplements called prebiotics. Prebiotics include different types of dietary fibre that we cannot digest but the bacteria can. There are also foods called probiotics that have high concentrations of good bacteria (e.g. yogurt).

One of the good things that happens when we eat these prebiotics and probiotics is they can help keep our bones strong. Postmenopausal women are at an increased risk of developing osteoporosis and more than 40% of Australian women will experience a bone fracture in their lifetime. Animal studies suggest eating prebiotics or probiotics may keep bones healthy. The effect of these supplements on bone health in humans is less clear.

The main purpose of this study is to see if a capsule containing a mix of probiotics and a prebiotic called inulin – which is a type of soluble fibre – can slow or stop bone thinning in women who are in the early stage of menopause. However, while this is primarily a bone health study, the type of gut health we are investigating is also linked to a range of other health outcomes. So, we will also be looking at outcomes like memory and learning, muscle and fat mass, blood sugar, well-being, and general and mental health.

We will invite up to 160 women to participate. You are one of them because you are postmenopausal, and it has been at least one year since your last menstrual cycle.

Who is undertaking the project?

This trial is run by Professor John Hawley from the Mary MacKillop Institute of Health Research at the Australian Catholic University. Prof Lorentzon has a strong background in trials investigating the effect of different interventions on bone density and fracture risk, including probiotic supplement trials. The OsteoPreP researchers are a multidisciplinary team with areas of expertise in bone health, gut health, women's health, healthy ageing, cardiovascular health, cognitive functioning, exercise physiology, endocrinology, and nutrition.

What is the probiotic supplement being used in this study and where does in come from?
The probiotic supplement being used in this study is being provided by Pendulum Therapeutics in San Francisco. Pendulum Therapeutics is a commercial biotechnology company involved in improving health through products targeting the human microbiome (gut bacteria). With regard to the OsteoPreP Study, Pendulum Therapeutics is responsible for providing the capsules (active and placebo) and funding some of the glucose-related measures.

The capsules contain 5 unique strains of bacteria that help digest dietary fibre and produce butyrate, a key molecule in bone health. They also contain inulin, a prebiotic complex carbohydrate that will be fermented by the bacteria strains upon absorption. You are asked to take one capsule within 30 minutes of your morning meal, and one capsule within 30 minutes of your evening meal, daily for 12 months.

Are there any risks associated with participating in this project?
You may have none, some or all of the effects listed below, and they may be mild, moderate, or severe. If you have any of these side effects, or are worried about them, please talk with someone from the research team.

<u>Risks of supplementation</u>

We expect you to experience minimal to no side effects from the supplements. Previous studies using similar supplements and dosing amounts have not indicated any severe side effects and most indicate no side effects. Possible side effects include transient mild nausea, loose stools, and diarrhea during the initial 3–5 days of use.

<u>Risk of bone mineral density scans</u>

This research project involves exposure to a very small amount of radiation. As part of everyday living, everyone is exposed to naturally occurring background radiation and receives a dose of about 2 millisieverts (mSv) each year. You will undergo specialised X-ray scans involving low amounts of radiation. These scans will include dual-energy X-ray absorptiometry (DXA) of your whole body, hip, and lumbar spine (lower back) and high resolution peripheral quantitative computed tomography (HR-pQCT) scans of your tibia (lower leg) and radius (forearm) at the standard site and at 30% of the bone length of the tibia and radius. The total effective radiation dose for the study is less than 0.2 mSv which is categorised as minimal risk; no harmful effects of radiation have been seen at this level of radiation.

The DXA scan will tell us if you have osteoporosis. If this happens, we will inform you immediately and you will be advised to refer to your doctor for further evaluation and treatment. The HR-pQCT scan is for research purposes only and is not intended for diagnosis.

<u>Risk of blood draw</u>

Risks associated with drawing blood from the arm include momentary discomfort and/or bruising. Infection, excess bleeding, clotting, or fainting are also possible, although unlikely. You will be asked to report any side effects to the study team immediately so we can assist you. The noted side effects are readily treatable. To minimise the risk of side effects, blood will be drawn by a trained professional, using sterile techniques. You will have your blood drawn at three testing visits.

<u>Risk of continuous glucose monitor</u>

There is a risk of minor discomfort as the device is inserted under the skin, and some ongoing irritation at the site of insertion while wearing the device.

What will I be asked to do?

Participation in this study will involve taking two capsules a day (one capsule with breakfast and one capsule with dinner) for one year. The capsules will either be the probiotic plus prebiotic combination or a placebo capsule. Over one year, you will be asked to attend at least five visits at the **Daniel Mannix Building, Australian Catholic University (ACU), 20 Brunswick Street, Fitzroy** (please call 0484 190 073 when you are out the front of the building so one of the research team can come and meet you, see map below).

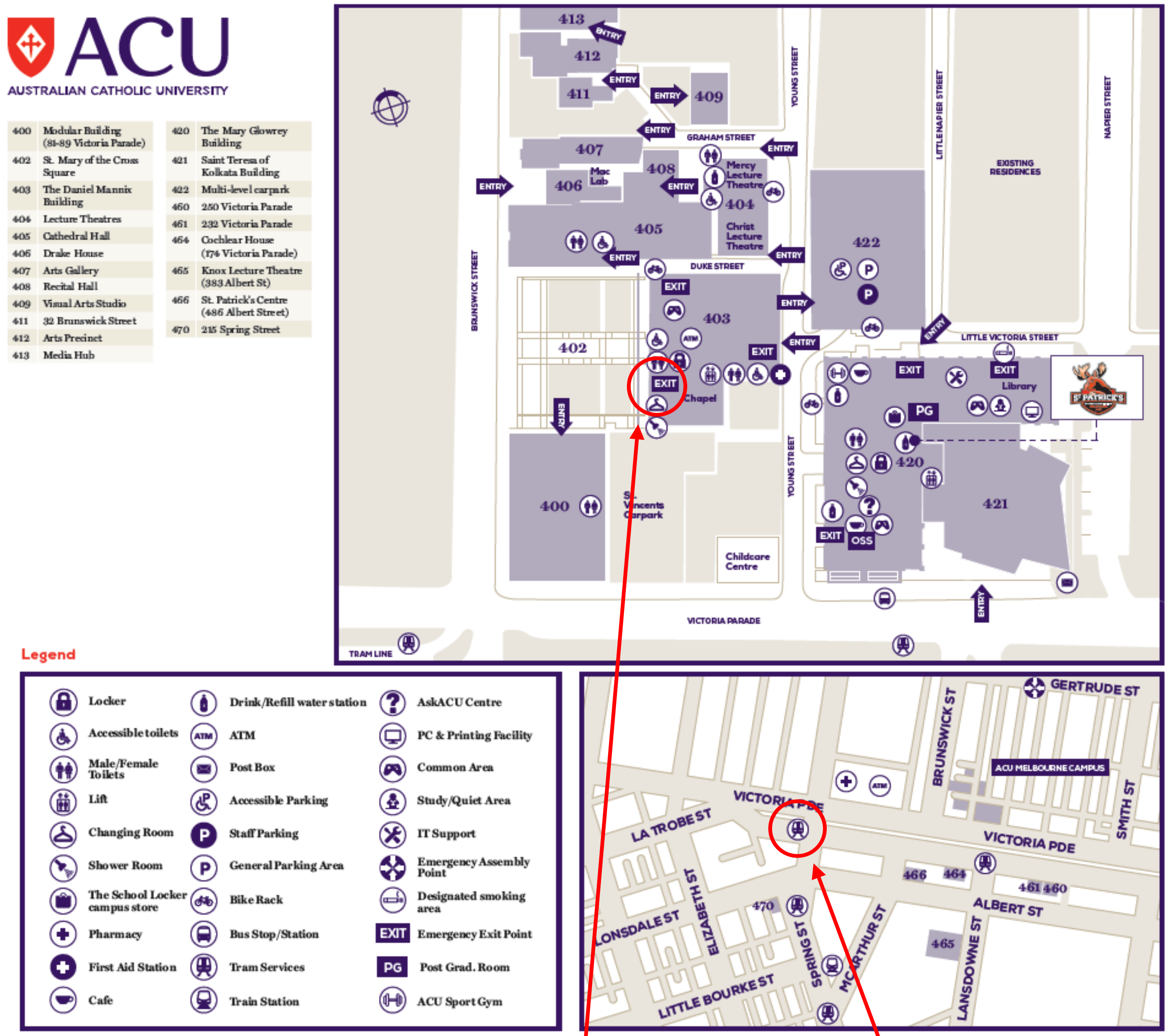

Entry to the Daniel Mannix Building

Tram stop #12 -St Vincent's Plaza/Victoria Pde (Fitzroy)

Most of the visits will be less than three hours, and the longest visit will be the 12-month visit at approximately 4 hours. Your initial visit will be a screening visit. During this visit we will ask you questions about your health and check your eligibility by performing a finger prick blood test, measuring your blood pressure, checking your medical history, and performing a bone density scan. If you are enrolled in the study, we will see you about two weeks later for your baseline visit, which is when you will start taking the supplement. We will see you again after 6 and 12 months and contact you via the phone and email after 3 and 9 months. There is also a final data collection point, two weeks after you have finished taking the supplements where you will be asked to drop off a stool sample to the research team at the Daniel Mannix Building of ACU in Fitzroy.

The table below outlines what is happening at each visit and how much time we anticipate everything to take. **Please note, due to bone density scanning scheduling, DXA and HR-pQCT scans may be scheduled together in a separate extra visit at screening and at the 12-month visit.**

**Assessments**	**Visit**	Telephone/ online	Screening	Baseline	3-month (telephone/ online	6-month	9-month (telephone/ online)	12-month	12-month + 2 week
	**Time required**	~10 mins	~150 mins	~150 mins	~20 mins	~180 mins	~20 mins	~240 mins	~15 mins
Informed written consent	10 mins								
Finger prick for HbA1c	5 mins								
Demographics/medical history	20 mins								
Blood pressure	20 mins								
Physical measures	10 mins								
Cogstate (memory tests)	25 mins		Practice						
Fasting blood draw	45 mins								
DXA scan	30 mins								
HR-pQCT scan	60 mins								
Questionnaire (completed online beforehand)	60 mins			Completed beforehand	GSRS only	Completed beforehand	GSRS only	Completed beforehand	Exit survey only
Dispense supplements	10 mins								
Physical activity (accelerometer)	10 mins								
Continuous Glucose Monitor	10 mins								
Stool sample	10 mins								

Abbreviations: HbA1c : glycated haemoglobin; DXA: Dual-energy X-ray Absorptiometry; HR-pQCT: High Resolution peripheral Quantitative Computed Tomography; GSRS: Gastrointestinal Symptom Rating Scale

COVID-19 Screening

Twenty-four hours before coming in for a scheduled visit, you will be emailed a link to a COVID-19 screening declaration form. If you have any of the symptoms, please inform the researchers and your visit will be re-scheduled.

Inclusion Exclusion Criteria

**OsteoPreP Study**	
**Inclusion criteria**	**Exclusion criteria**
— Postmenopausal women, 40-65 years old<br>— At least one year from their final menses<br>— Stated availability throughout the entire study period<br>— Agree to take two capsules daily for 12 months<br>— Agree to be randomly allocated to either the active group or the placebo group<br>— Self-identify as Caucasian<br>— Up to date Covid 19 vaccination status<br>— Able to walk without the use of an aid<br>— Mental ability to understand and willingness to fulfil all the details of the protocol<br>— Signed informed consent	— Diagnosis of osteoporosis<br>— T-score of -2.5 or less at the femoral neck or lumbar spine (L1-L4) on the DXA scan at screening visit<br>— Untreated hyperthyroidism<br>— Rheumatoid arthritis<br>— Diagnosed with a disease-causing secondary osteoporosis or malabsorption: chronic obstructive pulmonary disease, inflammatory bowel disease, celiac disease, type1/type2 diabetes, or chronic liver disease<br>— Bariatric surgery<br>— HbA1c ≥6.5% at screening visit<br>— Blood pressure at screening visit of systolic >180 mmHg and/or diastolic >120 mmHg<br>— Recently diagnosed malignancy (within the last 5 years)<br>— Current or recent oral corticosteroid use (any dose within the last 3 months, or 5mg of Prednisolone (or equivalent) or a higher daily dose for 14 days or more 3-12 months prior to screening)<br>— Use of antiresorptive therapy, including systemic hormone replacement therapy, bisphosphonates, RANK ligand inhibitor, selective estrogen receptor modulators, strontium ranelate (current or during the last 3 years)<br>— Use of teriparatide (current or during the last 3 years)<br>— Participation in other clinical intervention trials<br>— Antibiotics treatment 2 months prior to inclusion<br>— Unwilling to cease taking other probiotic or prebiotic supplements (current use)

Screening Visits (Assessments/Scans)

You will be asked to attend a screening visit. Here, you will be asked to sign an informed consent form, complete a medical history questionnaire, and undergo a finger prick test to assess your blood glucose levels. We will also perform a resting blood pressure assessment. The test will be repeated two to three times to ensure an accurate reading. At some point during this visit, you will be asked to complete a familiarisation with a memory test, Cogstate. Your performance on this test will be recorded, but it will only serve as a practice ahead of your baseline visit. Before you leave, we will also provide you with devices

to measure your physical activity (accelerometer) and your blood glucose as well as stool sample collection kits and detailed instructions for the use of these.

- Your *physical activity* will be measured using a small electronic device (ActiGraph GTX3) that you attach to yourself at the hip and wear over a period of ten days. You will be given a belt to attach it at the hip and it is to be taken off when you shower and when you sleep. You will return the device to the research team at your **baseline** visit.
- You will also be provided with a *blood glucose monitoring system* (Freestyle Libre Pro IQ) to take home and wear over a period of 10 days. The minimally invasive device will enable us to measure your glucose during your normal daily activities and while you sleep. The device is waterproof and is approximately the size of a 50-cent piece. The sensor is inserted rather painlessly into the back of the upper arm, which we will assist you with. This device is also returned to the research team at your **baseline** visit.
- For the *stool sample*, your kit will include a stool collector toilet attachment, gloves, two stool collection containers, some Ziplock sandwich bags, reusable gel ice pack, and a Thermabag for transport. You will be given four kits at the screening visit to cover the four time points that we would like samples from (baseline, 6 months, 12 months, and two weeks post intervention). You will be requested to collect a stool sample within 72 hours of each of these scheduled visits. We would like you to return your first stool sample during the baseline visit. The first movement of the day is preferable. To collect the sample, you will place a special collection bag into the bowl of your toilet before you use the toilet. When finished you will use the lid collection scoops to collect one sample from the first part of the stool. You will then take a second scoop from another part of the stool sample, seal the containers and place both samples in a plastic bag. The Ziplock bag can then be stored in your freezer (with the provided ice pack) for up to 3 days before your baseline visit. On the day of the baseline visit, you will be asked to take your samples out of the freezer, wrap them in the ice pack and place them in the Thermabag during your travel to the visit site. After collection, your de-identified stool samples will be sent to a collaborator for content analysis. Any remaining stool samples will be returned to ACU at the completion of the study.

At the screening visit, you will also undergo a DXA scan to ensure you do not have osteoporosis. No special preparations are needed for a DXA scan. You will need to remove any clothes that have metal fasteners, such as zips or hooks; in some cases you may need to wear a gown. You will be asked to lie on your back on a flat, open X-ray table and you will need to keep very still during the scan. As the scanning arm is moved slowly over your body, a narrow beam of low-dose X-rays will be passed through your body to measure bone density in the centre of the skeleton. The results of this test will be assessed and if you have no signs of osteoporosis, you will be able to participate in the study. You will then also undergo a second bone scan – HR-pQCT. As with the DXA, no special preparations are needed for a HR-pQCT scan. You are being scanned at the lower arm and lower leg, which will need to be free of any metal (jewellery, for example). You will be asked to place your arm/leg into the scanner. Once again you will need to remain very still during the scan and the part of your body being scanned will be exposed to a low dose X-ray.

If after the finger prick blood test, blood pressure assessment or DXA scan you are identified as having abnormal results, we will let you know of this immediately, provide you with some information about what this means, and ask you to see your General Practitioner. Unfortunately, you will not be able to continue your participation in the OsteoPreP study if this is the case.

<u>Baseline Visit</u>

If you screen into the study, you will be booked in for a **baseline visit**. Ahead of the baseline visit, you will be emailed secure links to the questionnaires associated with the study. We ask that you complete these

questionnaires prior to attending the baseline visit. Please know your responses will be saved in a secure system which is only accessible to the research team. You will be asked to fast before this visit (see below for details). When you first arrive, we will collect your accelerometer and blood glucose monitoring device and the freezer bag stool sample and wil measure your blood pressure. We will then escort you down to St Vincent's Hospital Pathology department where they will draw some of your blood.

For this **fasting blood sample**, please ensure that you have had nothing to eat or drink (water is okay) for 8-12 hours prior to your fasting blood test. Please ensure you drink plenty of water to stay well hydrated and take any medication as normal. A trained professional will insert a cannula into your arm and then proceed to fill several tubes with your blood (50mL in total). The cannula is then removed, and pressure is applied to the insertion point to stop the bleeding with a bandage. A portion of your de-identified collected blood may be transferred to the Victorian Cancer Biobank for processing and storage but will be returned to ACU at the completion of the study.

After the blood draw is completed, we will have you perform a grip strength test that will require you to squeeze a specialised hand grip device with maximum effort 2-3 times per hand. We will then also quickly check that all questionnaire components are completed. The questionnaires cover a diverse range of topics including quality of life, diet over the previous month, physical activity over the last 7 days, depression/anxiety/stress, and gastrointestinal symptoms, plus the Cogstate memory test completed on a laptop.

At the end of the baseline assessment, you will be given a seven-month supply of capsules and instructed to take one capsule twice daily within 30 minutes of the morning and evening meal. You will also be asked to track your consumption daily, as well as any side effects you might be experiencing.

<u>Three-Month Timepoint</u>
After 3 months we will contact you via the telephone to check you are taking the supplements, to ask you about any adverse events, and to ask you to complete an online survey to record if you have had any gastrointestinal side effects (bloating, or upset stomach, for example).

<u>Six-Month Visit</u>
Just ahead of the 6-month visit you will be sent a link with the same questionnaires you completed at baseline. Within 72 hours of this visit, you will be asked to collect a stool sample. For the visit, you will be asked to visit the Daniel Mannix Building of ACU in Fitzroy in a **fasted state**. After collecting your stool sample and supplement containers (included any unconsumed supplements), we will check you are taking the supplements, record if you have had any side effects and give you another 6-month supply of supplements. You will also be asked to complete the Cogstate memory test. We will take another fasting blood sample, perform a finger prick test, and conduct a blood pressure and grip strength assessment. During this visit you will be fitted out with the accelerometer and continuous glucose monitor. You will be asked to wear these devices for a period of 10 days and return the devices by registered post (pre-labelled envelope will be provided).

<u>Nine-Month Timepoint</u>
The 9-month timepoint contact will be identical to the 3-month timepoint, where we will contact you via the telephone to check you are taking the supplements and ask you about adverse events, as well as complete the online survey to record any gastrointestinal side effects that you may have experienced.

<u>Twelve-Month Visit</u>

Just ahead of the 12-month visit you will be sent a link with the same questionnaires you completed at baseline. Within 72 hours of your scheduled 12-month visit, you will be required to collect a stool sample. For the visit, you will once again return **fasted** to the Daniel Mannix Building of ACU in Fitzroy and undergo the same assessments that you did at the baseline visit in addition to a DXA and HR-pQCT scan. You will also be fitted with the continuous glucose monitor and accelerometer to wear for 10-days after the 12-month visit. After the 10 days you will stop taking the supplements. You will be asked to collect a final stool sample 14-17 days after you have stopped taking the supplement. Following this, you will come in for your final study visit.

Two-Weeks After Stopping Supplementation (final study visit)
Approximately two weeks after you stop taking the supplements (14-17 days) you will be asked to provide a final stool sample so we can see what happens to your gut bacteria. You will be asked to bring this stool sample, the continuous glucose monitor, the accelerometer, the compliance calendars, and the supplement containers (and any remaining supplement) to the Daniel Mannix Building of ACU in Fitzroy. We can meet you at your vehicle to collect these from you. We will then provide you with your study reimbursement gift card.

What are the benefits of the research project?
We cannot guarantee that you will receive any benefits from this research; however, a possible benefit may be improved bone health. We hope that in the future, the information gathered from this study will be used to inform new therapeutics that improve bone health and aid in the prevention of osteoporosis.

You will be reimbursed $200 at the completion of all testing visits to cover your expenses and transport related to the study.

Can I withdraw from the study?
Participation in this study is completely voluntary. You are not under any obligation to participate. If you agree to participate, you can withdraw from the study at any time by filling out the withdrawal form (via the attached withdrawal form or online) without any adverse consequences. If you decide to withdraw your consent during the project, the study team will not collect any further personal information, samples, or data from you. You should be aware that data collected up to the time you withdraw will form part of the research project results unless you specify otherwise. You will be given the opportunity to specify what you want to happen to your personal information, samples, and data when you complete the withdrawal form. You are also able to withdraw your data and/or samples at any time after the conclusion of the study (if still within the data and sample retention periods).

Will anyone else know the results of the project?
Study results will be published in scientific journals on a group level (all participant details will be de-identified) and disseminated to the public through press-releases and popular science articles. While this is primarily a bone health study, we are also looking at a range of other health outcomes and therefore, we aim to publish and disseminate the de-identified results of these as well, either in relation to bone health or separately if appropriate.

All data collected will be assigned a coded participant ID which is free of any personal identifying information. Any personal information obtained during the study will be stored separate to research data collection sheets and kept confidential. Access to participant personal information will be restricted to the research team only and stored in a password-locked computer.

If you consent to your data being stored for future closely related research, your de-identified information could be shared with other researchers, either those named within this document or future collaborators. Also, your de-identified data may be used by external data collection providers (Cogstate and Victorian Cancer Council Food Frequency Questionnaire data only) for purposes of their own research and development. Your personal and health data will be stored for at least 20 years post study completion and your blood and stool samples will be stored for at least 10 years post study completion.

In accordance with relevant Australian and/or Victorian privacy and other relevant laws you have the right to access the information collected and stored by the researchers about you. You also have the right to request that any information with which you disagree to be corrected. Please contact one of the researchers named below if you would like to access your information.

Will I be able to find out the results of the project?

Results on a group level will be made available on a study web page. After study completion, you may be invited to a study meeting to learn about the study findings.

Who do I contact if I have questions about the project?

If you require further information or if you have any problems concerning this project (for example, any side effects), you can contact any of the team members:

Principle researcher: Prof John Hawley
Office phone: 03 9953 3552
Email: john.hawley@acu.edu.au

OsteoPreP research team:
Phone: 0484 190 073
Email: OsteoPreP@acu.edu.au

Conflict of interest statement

The researchers declare that this study will be conducted such that any commercial or financial relationships will not pose any conflict of interest. Pendulum Therapeutics will not be involved in the conduct of this study.

What if I have a complaint or any concerns?

The study has been reviewed by the Human Research Ethics Committee at Australian Catholic University (review number 2012-122HC). If you have any complaints or concerns about the conduct of the project, you may contact The Research Ethics and Integrity Manager.

The Research Ethics and Integrity Manager
C/O Office of the Deputy Vice Chancellor (Research)
Australian Catholic University
North Sydney Campus
PO Box 968
NORTH SYDNEY, NSW 2059
Phone: 02 9739 2519
Email: resethics.manager@acu.edu.au

Any complaint or concern will be treated in confidence and fully investigated. You will be informed of the outcome.

I want to participate! How do I sign up?

If you wish to participate, please sign the attached consent form.

Consent Form - *Adult providing own consent*

**Title**	OsteoPreP: The effect of probiotic supplementation on bone, muscle, and glucose metabolism in postmenopausal women: A randomized placebo-controlled trial
**Short Title**	OsteoPreP
**Application Number**	2021-122HC
**Project Sponsor**	Australian Catholic University
**Principal Investigator**	Professor John Hawley
**Site Principal Investigator**	Professor John Hawley
**Associate Investigators**	Professor Mattias Lorentzon, PhD, Dr Andrew Garnham, MD, Dr Anoohya Gandham, PhD, Ms Alisa Turbić, Ms Marion MacRae and Ms Hadeel Nassar

<u>Consent Agreement</u>

☐ I have read the Participant Information Sheet, or someone has read it to me in a language that I understand, and I agree to participate in the study:

1. I understand the purposes, procedures and risks of the research described in the project.
2. I have had an opportunity to ask questions and I am satisfied with the answers I have received.
3. I freely agree to participate in this research project as described and understand that I am free to withdraw at any time during the study without affecting my future health care.
4. I understand that I will be given a signed copy of this document to keep.

☐ I consent to the storage and use of the de-identified personal and health information, and blood and stool samples taken from me, as described in the relevant section of the Participant Information Sheet, for:

a) This specific research project.

b) For use by Pendulum Therapeutics who may use my de-identified personal and health information, and stool and blood related data for the research and development of an associated commercial product.

c) For use by a commercial provider of metabolomics to undertake a comprehensive analysis of the small molecules contained in my blood.

d) For use by a commercial provider of genomics services (for example, Novogene) to undertake a comprehensive analysis of the genetic content of the bacteria in my stool.

☐ I understand that unless I complete the withdrawal form, my data and samples will form part of the research.

☐ I consent to the storage and use of the de-identified personal and health information, and blood and stool samples for use in other research that is closely related to this research study (i.e. for future research into bone health, gut health, women's health, healthy ageing, endocrinology, and nutrition). This may include research involving animal subjects.

☐ I consent to my contact details being added to the Mary MacKillop Institute for Health Research (MMIHR) participant database to be contacted for future MMIHR research projects.

Please sign:

Name of Participant (please print) ______________________________
Signature ____________________ Date ____________________

Declaration by Study Staff/Senior Researcher†

I have given a verbal explanation of the research project; its procedures and risks and I believe that the participant has understood that explanation.

Name of Study Staff/<br>Senior Researcher† (please print) ______________________________
Signature ____________________ Date ____________________

† Dr Anoohya Gandham, Ms Alisa Turbic, Ms Marion MacRae, or Ms Hadeel Nassar must provide the explanation of, and information concerning, the research project.

Note: All parties signing the consent section must date their own signature.

Release of medical information

☐ In the case of an adverse event during the study period, I consent to the study site doctor contacting my GP/medical specialist to obtain my medical information for follow up if required.

Name of GP/medical specialist____

Address____

Contact number____

Name of Participant (please print) ____<br>Signature ________________________________ Date ________________________________

Form for Withdrawal of Participation

**Title**	OsteoPreP: The effect of probiotic supplementation on bone, muscle, and glucose metabolism in postmenopausal women: A randomized placebo-controlled trial
**Short Title**	OsteoPreP
**Application Number**	2021-122HC
**Project Sponsor**	Australian Catholic University
**Principal Investigator**	Professor John Hawley
**Site Principal Investigator**	Professor John Hawley
**Associate Investigators**	Professor Mattias Lorentzon, PhD, Dr Andrew Garnham, MD, Dr Anoohya Gandham, PhD, Ms Alisa Turbic, Ms Marion MacRae and Ms Hadeel Nassar

Declaration by Participant

I wish to withdraw from participation in the above research project and understand that such withdrawal will not affect my routine treatment, my relationship with those treating me or my relationship with the Australian Catholic University.

Regarding my data and samples collected thus far:

Please note, if you do not check any of the below boxes, your data and samples will be used as per your previous consent.

- ☐ I wish to leave any personal and health data collected for use as per my previous consent
- ☐ I wish to leave any blood and stool samples collected for use as per my previous consent

- ☐ I wish any personal and health information collected to be destroyed or deleted*
- ☐ I wish any blood and stool samples collected to be destroyed and any related data deleted*

*Hard copy data will be shredded, biological samples will be destroyed, electronic data will be deleted

Name of Participant (please print) ______________________________

Signature ______________________ Date ______________________

Participant Emergency Contact Numbers and Support Information

Urgent Medical Attention

Please call 000 (triple zero)

Melbourne Metropolitan Public Hospital Information

Click on the below link for more information about Melbourne Metropolitan Public Hospitals

https://www2.health.vic.gov.au/hospitals-and-health-services/public-hospitals-victoria

Melbourne Metropolitan Public Hospital Emergency Departments

If you require emergency medical attention please refer to the map of Metropolitan Melbourne's Public Hospitals on the next page to find out your closest emergency department.

Nurse on Call

A Victorian Government health initiative, this is a phone service that provides immediate, health advice and information from a registered nurse, 24 hours a day, 7 days a week

1300 60 60 24 - Nurse on Call

24-Hour Helpline and Online Support

Lifeline - 13 11 14 (confidential 24/7 counselling and referrals)

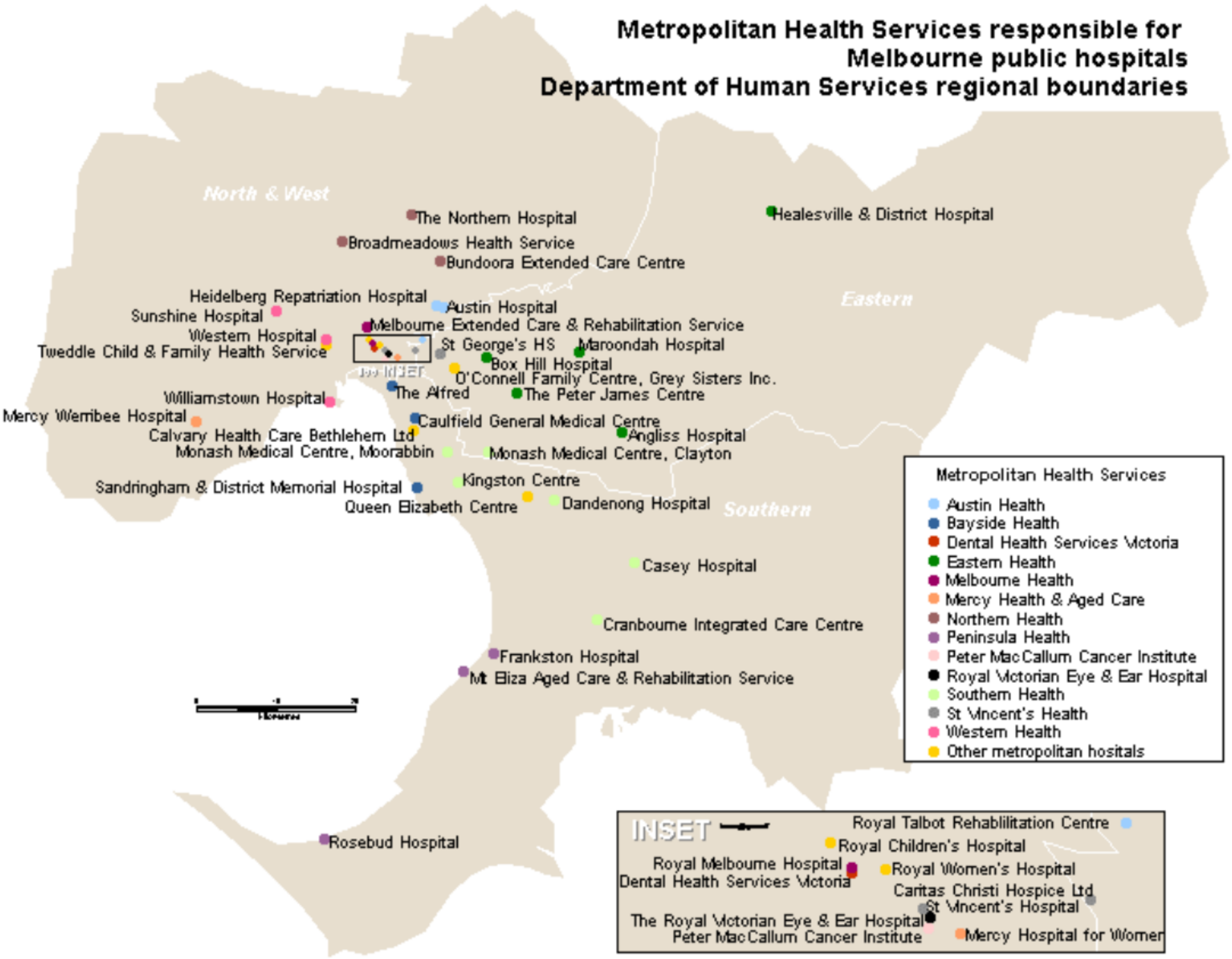
Metropolitan Health Services responsible for
Melbourne public hospitals
Department of Human Services regional boundaries
North & West
Eastern
Southern
The Northern Hospital
Broadmeadows Health Service
Bundoora Extended Care Centre
Healesville & District Hospital
Heidelberg Repatriation Hospital
Sunshine Hospital
Austin Hospital
Melbourne Extended Care & Rehabilitation Service
Western Hospital
Tweddle Child & Family Health Service
St George's HS
Maroondah Hospital
Box Hill Hospital
O'Connell Family Centre, Grey Sisters Inc.
The Alfred
The Peter James Centre
Williamstown Hospital
Mercy Werribee Hospital
Caulfield General Medical Centre
Calvary Health Care Bethlehem Ltd
Angliss Hospital
Monash Medical Centre, Moorabbin
Monash Medical Centre, Clayton
Sandringham & District Memorial Hospital
Kingston Centre
Queen Elizabeth Centre
Dandenong Hospital
Casey Hospital
Cranbourne Integrated Care Centre
Frankston Hospital
Mt Eliza Aged Care & Rehabilitation Service
Rosebud Hospital
Metropolitan Health Services
Austin Health
Bayside Health
Dental Health Services Victoria
Eastern Health
Melbourne Health
Mercy Health & Aged Care
Northern Health
Peninsula Health
Peter MacCallum Cancer Institute
Royal Victorian Eye & Ear Hospital
Southern Health
St Vincent's Health
Western Health
Other metropolitan hositals
INSET
Royal Talbot Rehablilitation Centre
Royal Children's Hospital
Royal Melbourne Hospital
Royal Women's Hospital
Dental Health Services Victoria
Caritas Christi Hospice Ltd
St Vincent's Hospital
The Royal Victorian Eye & Ear Hospital
Peter MacCallum Cancer Institute
Mercy Hospital for Women
