# Supplementary material for: Effects of Synbiotic Supplementation on Bone and Metabolic Health in Caucasian Postmenopausal Women: Rationale and Design of the OsteoPreP Trial
Source: Nutrients. 2024 Dec 6;16(23):4219. doi: 10.3390/nu16234219 (PMC11644401; doi:10.3390/nu16234219)
Supplement: Supplementary file 1 [file nutrients-16-04219-s001.zip › nutrients-3338017-supplementary/Supplementary files/Supplementary file S8.pdf]

ACU
AUSTRALIAN CATHOLIC UNIVERSITY

# PARTICIPANT INFORMATION LETTER

**PROJECT TITLE:** OsteoPreP mechanistic sub-study: The effect of probiotic supplementation on bone, muscle, and glucose metabolism in postmenopausal women: A randomized placebo-controlled trial

**APPLICATION NUMBER: 2021-122HC**
**PRINCIPAL INVESTIGATOR:** Prof Mattias Lorentzon

Dear Participant,

You are invited to participate in the research project described below.

**Please note, participation in this sub-study is OPTIONAL and will not impact your participation in the Main OsteoPreP study in any way.**

***What is the project about?***
The primary purpose of the **main OsteoPreP Study** is to see if a probiotic supplement can slow or stop bone thinning in women who are going through early post-menopause. The purpose of **this subgroup study** is to investigate if the probiotic intervention also affects your muscle tissue, and your ability to process glucose.. The muscles form part of the musculoskeletal system and early post-menopausal women experience a period of rapid muscle mass loss too, called Sarcopenia. Sarcopenia has also been linked to insulin resistance. If the supplements do have a positive effect on the bone, muscle, and glucose, it will be important to understand what happened to facilitate those changes. We will invite 30 women to participate in this additional subgroup.

***Who is undertaking the project?***
This trial is run by Professor Mattias Lorentzon from the Bone Health and Fractures Research Program at the Australian Catholic University and the OsteoPreP research team.

***Are there any risks associated with participating in this project?***
You may have none, some or all the effects listed below, and they may be mild, moderate, or severe. If you have any of these side effects, or are worried about them, please talk with someone from the research team.

<u>Risk of blood draw and muscle tissue sampling</u>
Risks associated with drawing blood from the arm include momentary discomfort and/or bruising. Infection, excess bleeding, clotting, or fainting are also possible, although unlikely. Blood will be drawn by a trained professional, using sterile techniques to minimize these risks.

Having a muscle tissue sample taken can also cause some discomfort, bruising, minor infection, or bleeding. All of these are easily treatable. You will be monitored during the procedure and someone from the study team will contact you after the procedure to ensure that you are recovering as expected.

***What will I be asked to do?***
Participation in this subgroup will involve completing additional assessments to those included in the main OsteoPreP study at the baseline, 6-month and 12-month time points. You are still able to participate in the OsteoPreP study if you do not want to take part in these additional assessments.

If you would like to take part in these additional measures, you will be asked to attend 3 additional study visits in total. These additional visits will be for a muscle biopsy and oral glucose tolerance test (one at baseline, 6 months, and 12 months) each lasting between 2 and 3 hours. These additional visits will be identical at the three time points. Details of the additional assessments are included below.

Muscle biopsy and oral glucose tolerance test visit (~2.5 hours)

- This visit will occur at the **ACU Exercise Laboratory, Level 1, Daniel Mannix Building, 15 Young Street, Fitzroy (see map below).**

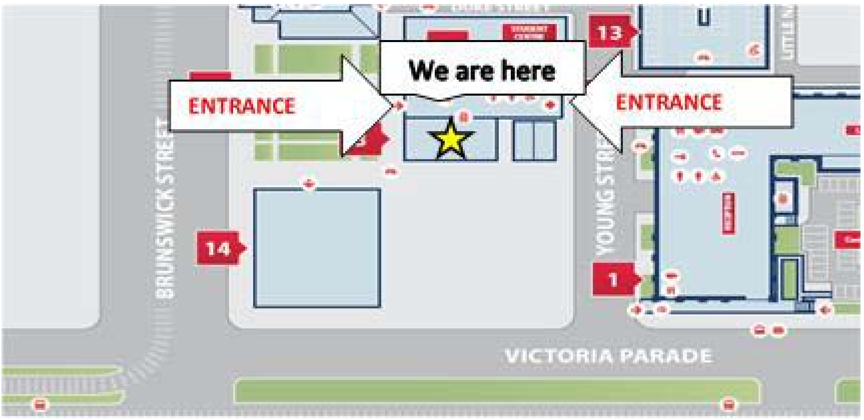


- We need you to fast from midnight the night before your muscle biopsy visit which will be scheduled early in the morning. The **muscle biopsy** will be performed by an experienced doctor who will give you a local anaesthetic in your thigh and while lying down, a small incision will be made into the thigh muscle and a small tissue sample will be taken out. This is an outpatient procedure and you will not require any stitches.

- The **oral glucose tolerance test** will be completed on the same day. A needle with plastic cannula line will be inserted by a trained professional to allow blood to be taken (42 mL total). After an initial fasting sample is taken, you will be asked to drink 8 ounces of a syrupy glucose drink (75g sugar) and following this, a blood sample will be drawn every 30 min for two hours.

***What are the benefits of the research project?***
We cannot guarantee that you will receive any benefits from this research; however, a possible benefit may be improved musculoskeletal health.

Upon completion of the muscle biopsy, you will be reimbursed $100 for each sample collection point (0, 6, and 12 months). This $300 will be in addition to the standard $100 for the main study.

***Can I withdraw from the study?***

Participation in this sub study is voluntary. If you do not wish to take part, you do not have to, and this will not affect your participation in the regular OsteoPreP Study. If you decide to take part and later change your mind, you are free to withdraw from these additional assessments at any stage, and this will not impact your participation in the regular study. You can withdraw from the sub study at any time by filling out the withdrawal form (via the attached withdrawal form or online). If you decide to withdraw your consent during the project, the study team will not collect any further sub study related personal information, samples, or data from you. You should be aware that data collected up to the time you withdraw will form part of the research project results unless you specify otherwise. You will be given the opportunity to specify what you want to happen to your personal information, samples, and data when you complete the withdrawal form. You are also able to withdraw your data and/or samples at any time after the conclusion of the study (if still within the data and sample retention periods).

If you do decide to take part, you will be asked to sign this Participant Information and Consent Form, which you will be given a copy to keep. Your decision whether or not to take part, or to take part and then withdraw, will not affect your relationship with the Australian Catholic University.

***Will anyone else know the results of the project?***

Study results will be published in scientific journals on a group level (all participant details will be de-identified) and disseminated to the public through press-releases and popular science articles. While this is primarily a bone health study, we are also looking at a range of other health outcomes and therefore, we aim to publish and disseminate the deidentified results of these as well, either in relation to bone health or separately if appropriate.

All data collected will be assigned a coded participant ID which is free of any personal identifying information. Any personal information obtained during the study will be stored separate to research data collection sheets and kept confidential. Access to participant personal information will be restricted to the research team only and stored in a password locked computer.

If you consent for your data to be stored for future closely related research, your deidentified information could be shared with other researchers, either those named within this document or future collaborators. Also, your deidentified data may be used by external data collection providers for purposes of their own research and development. Your personal and health data will be stored for at least 20 years post study completion and your blood and muscle samples will be stored for at least 10 years post study completion.

In accordance with relevant Australian and/or Victorian privacy and other relevant laws you have the right to access the information collected and stored by the researchers about you. You also have the right to request that any information with which you disagree to be corrected. Please contact one of the researchers named below if you would like to access your information.

***Will I be able to find out the results of the project?***

Results on a group level will also be made available on a study web page. After study completion, you may be invited to a study meeting to learn about the study findings.

***Who do I contact if I have questions about the project?***

If you require further information or if you have any problems concerning this project (for example, any side effects), you can contact any of the team members:

Principle researcher: Prof Mattias Lorentzon
Office phone: 03 9230 8056
Email: Mattias.Lorentzon@acu.edu.au

Clinical Trial Project Coordinator: Research Team member 1
Office phone: 03 9230 ####
Email: Research.Teammember1@acu.edu.au

***Conflict of interest statement***
*The researchers declare that this study will be conducted such that any commercial or financial relationships will not pose any conflict of interest. Pendulum Therapeutics will not be involved in the conduct of this study.*

***What if I have a complaint or any concerns?***
The study has been reviewed by the Human Research Ethics Committee at Australian Catholic University (review number 2021-122HC). If you have any complaints or concerns about the conduct of the project, you may contact The Research Ethics and Integrity Manager.

The Research Ethics and Integrity Manager
C/O Office of the Deputy Vice Chancellor (Research)
Australian Catholic University
North Sydney Campus
PO Box 968
NORTH SYDNEY, NSW 2059
Ph.: 02 9739 2519
Email: resethics.manager@acu.edu.au

Any complaint or concern will be treated in confidence and fully investigated. You will be informed of the outcome.

***I want to participate! How do I sign up?***
If you wish to participate, please sign the attached consent form.

# **Consent Form -** *Adult providing own consent*

| | |
|---|---|
| **Title** | OsteoPreP Mechanistic Investigations Sub-study: The effect of probiotic supplementation on bone, muscle, and glucose metabolism in postmenopausal women: A randomized placebo-controlled trial |
| **Short Title** | OsteoPreP – Mechanistic Investigations Sub-study |
| **Application Number** | 2021-122HC |
| **Project Sponsor** | Australian Catholic University |
| **Principal Investigator** | Professor Mattias Lorentzon |
| **Site Principal Investigator** | Professor Liesbeth Vandenput |
| **Associate Investigators** | Dr Claus Christophersen, PhD, Professor John Hawley, PhD, A/Prof Francine Marques, PhD, Dr Andrew Garnham, MD, and Ms Anoohya Gandham. |

**Consent Agreement**

☐ I have read the Participant Information Sheet, or someone has read it to me in a language that I understand, and I agree to participate in the sub study:

1. I understand that participation in the sub study is optional.
2. I understand the purposes, procedures and risks of the research described in the project.
3. I have had an opportunity to ask questions and I am satisfied with the answers I have received.
4. I freely agree to participate in this sub study research project as described and understand that I am free to withdraw at any time during the sub-study without affecting my participation in the main study or future health care.
5. I understand that I will be given a signed copy of this document to keep.

Below we will ask for your consent the additional sub study tests and procedures:

- Oral glucose tolerance test and analysis
- Muscle biopsy

By signing your consent to these additional tests and procedures, you are consenting to completing both components. These additional assessments will be conducted at 3 timepoints, the start of the study, 6 months, and 12 months.

☐ I consent  to the storage and use of the de-identified personal and health information, and muscle tissue and blood samples taken from me, as described in the relevant section of the Participant Information Sheet, for:

a) This specific research project.

b) For use by Pendulum Therapeutics who may use my de-identified personal and health information, and muscle tissue and blood sample related data, for the research and development of an associated commercial product.

☐ I understand that unless I complete the withdrawal from, my data and samples will form part of the research.

☐ I consent to the storage and use of the de-identified personal and health information, and muscle tissue and blood samples taken from me, for use in other research that is closely related to this research study (i.e. for future research into bone health, gut health, women's health, healthy ageing, endocrinology, and nutrition). This may include research involving animal studies.

There may be cultural considerations that could inform your decision to provide this unspecified consent. You may want to discuss the issue of tissue donation with those closest to you.

By signing this consent section, I agree to the use of my de-identified muscle biopsy and blood samples for testing, as outlined in the relevant Section of the Participant Information Sheet.

**Declaration by Participant – for participants who have read the information**

Name of Participant (please print) ______________________________________________

Signature ______________________________ Date ______________________________

**Declaration by Study Doctor/Senior Researcher†**

I have given a verbal explanation of the research project; its procedures and risks and I believe that the participant has understood that explanation.

Name of Study Staff/
Senior Researcher† (please print) ______________________________________________

Signature ______________________________ Date ______________________________

† Research Team member 1, Research Team member 2, or Research Team member 3 must provide the explanation of, and information concerning, the research project.

Note: All parties signing the consent section must date their own signature.

## Release of medical information

☐ In the case of an adverse event during the study period I consent to the study site doctor contacting my GP/medical specialist to obtain my medical information for follow up if required.

Name of GP/medical specialist_______________________________________________________

Address__________________________________________________________________________

Contact number___________________________________________________________________

Name of Participant (please print) ____________________________________________________

Signature _________________________________ Date _________________________________

# Form for Withdrawal of Participation

| | |
|---|---|
| **Title** | OsteoPreP Mechanistic Sub-study: The effect of probiotic supplementation on bone, muscle, and glucose metabolism in postmenopausal women: A randomized placebo-controlled trial |
| **Short Title** | OsteoPreP – Mechanistic Sub-study |
| **Application Number** | 2021-122HC |
| **Project Sponsor** | Australian Catholic University |
| **Principal Investigator** | Professor Mattias Lorentzon |
| **Site Principal Investigator** | Professor Liesbeth Vandenput |
| **Associate Investigators** | Dr Claus Christophersen, PhD, Professor John Hawley, PhD, A/Prof Francine Marques, PhD, Dr Andrew Garnham, MD, and Ms Anoohya Gandham. |

**Declaration by Participant**

I wish to withdraw from participation in the above research project and understand that such withdrawal will not affect my routine treatment, my relationship with those treating me or my relationship with the Australian Catholic University.

Regarding my data and samples collected thus far:

Please note, if you do not check any of the below boxes your data and samples will be used as per your previous consent.

- ☐ I wish to leave any personal and health data collected for use as per my previous consent
- ☐ I wish to leave any blood and muscle samples collected for use as per my previous consent

- ☐ I wish any personal and health information collected to be destroyed or deleted*
- ☐ I wish any blood and muscle samples collected to be destroyed and any related data deleted*

*Hard copy data will be shredded, biological samples will be destroyed, electronic data will be deleted

Name of Participant (please print) ______________________________

Signature ______________________ Date ______________________
